# Efficacy of the Intervention Against the Stigmatization of Men With Eating Disorders in Primary Healthcare (iSMEsH): Results From a Randomized Waitlist‐Controlled Study

**DOI:** 10.1002/eat.70080

**Published:** 2026-03-09

**Authors:** Martin S. Lehe, Georg Halbeisen, Sabine Steins‐Loeber, Georgios Paslakis

**Affiliations:** ^1^ University Clinic for Psychosomatic Medicine and Psychotherapy, Medical Faculty, Campus East‐Westphalia Ruhr University Bochum Lübbecke Germany; ^2^ Department of Clinical Psychology and Psychotherapy University of Bamberg Bamberg Germany

**Keywords:** access to care, anti‐stigma, eating disorders, general practitioner, help seeking, male, medical staff, men, primary healthcare, stigma

## Abstract

**Objective:**

Eating disorders (EDs) in men are underdiagnosed and undertreated, partly due to stigma hindering help‐seeking. This randomized waitlist‐controlled study tested the efficacy of the iSMEsH online anti‐stigma intervention targeting German general practitioners (GPs) and medical students. The program aimed to reduce stigmatizing attitudes toward men with EDs and improve knowledge and self‐efficacy in managing ED symptoms.

**Method:**

A total of 292 participants (130 GPs, 162 medical students) were randomly assigned to immediate intervention or waitlist control. The intervention consisted of six video‐based, on‐demand modules co‐developed with men who have lived experience of an ED, combining education and contact‐based strategies. Outcomes included cognitive stigma (knowledge), affective stigma (biased attitudes), and behavioral stigma (treatment self‐efficacy), assessed at three timepoints.

**Results:**

The iSMEsH anti‐stigma intervention significantly increased knowledge of male‐specific ED presentations and enhanced treatment self‐efficacy in both GP and medical student populations. Effects on affective stigma were less consistent.

**Discussion:**

Findings support the efficacy of the iSMEsH anti‐stigma intervention in improving knowledge and treatment self‐efficacy regarding EDs in men among healthcare professionals. Effects on affective stigma were limited and may require longer follow‐up periods to be comprehensively captured. The intervention shows promise as a scalable tool to reduce stigma and improve care for men with EDs.

**Trial Registration:**

On July 1, 2024 (#181,415; https://aspredicted.org/tzds‐h5yq.pdf) and a study protocol is published under Lehe et al. (2025)

## Key Points/Summary

- Eating disorders (EDs) in men are frequently underdiagnosed and misunderstood, partly due to stigma, which also affects healthcare professionals and calls for effective anti‐stigma interventions.
- The iSMEsH online anti‐stigma intervention probed in this randomized controlled study significantly enhanced knowledge and self‐efficacy related to EDs in men among general practitioners (GPs) and medical students.
- The intervention showed less consistent effects on affective stigma, possibly due to short follow‐up periods.
- The training's brief, scalable, and on‐demand format highlights its potential for integration into medical education and GP practice to improve early detection and treatment access for men with EDs.

**Abbreviations**
ANOVA: Analysis of Variance
CG: Control Group
ED: Eating Disorder
EMM: Estimated Marginal Mean
GP: General Practitioner
GSE: General Self‐Efficacy Scale
IG: Intervention Group
ITT: Intention‐to‐treat
LMM: Linear Mixed Model
M: Mean
OMS‐HC: Opening Minds Stigma Scale for Health Care Providers
PP: Per‐protocol
SD: Standard Deviation

## 1 Introduction

Eating disorders (EDs) are a growing public health concern (Treasure et al. 2020) associated with substantial morbidity, mortality, and healthcare costs (Udo and Grilo 2019). Due to the frequent occurrence in adolescence, long duration of illness, and repeated inpatient stays, treatment costs are substantial (Krauth et al. 2002). Although women constitute the majority of ED cases across age groups, men may account for every fourth case (Ferrari et al. 2022). Men, however, remain underrepresented in ED research and care, for example, with one man with Anorexia nervosa or Bulimia nervosa for every 10–20 women in specialized treatment facilities (Flores et al. 2022; Statistisches Bundesamt 2021).

One factor contributing to men's underrepresentation is the so‐called “double stigmatization” (Mangweth‐Matzek 2022): Men may conceal having an ED, not only because they are ashamed of having a mental disorder, but also because having an ED, which is broadly considered a “women's disease”, and may conflict with traditional gender roles and internalized masculinity norms. Stigma includes linking characteristics to negative stereotypes or prejudice, often resulting in social devaluation and discrimination (Link and Phelan 2001). It manifests on the public level (public stigma)—through cognitive (e.g., stereotypes, misinformation), affective (e.g., prejudice, negative attitudes), and behavioral facets (e.g., discrimination)—and on the individual level as self‐stigmatization, reflecting the internalization of societal devaluation by affected individuals (Rüsch et al. 2005). Internalized stigma can reduce men's recognition and disclosure of ED symptoms and was linked to lower help‐seeking intentions for EDs in men in qualitative (Richardson and Paslakis 2021), quantitative (Griffiths et al. 2015; Lehe et al. 2024; Lehe, Halbeisen, Juergensen, et al. 2025), and mixed‐methods research (Bomben et al. 2022).

Men's self‐stigmatization makes it essential for healthcare professionals to regularly ask about EDs in men, address them, and refer men to appropriate treatment. At the same time, the stereotype of EDs as a “women's disease” also shapes the views and behaviors of medical staff (Brelet et al. 2021). According to a systematic review of men's experiences, men's EDs are frequently missed in healthcare, with reports of underrecognition, dismissal of complaints, and limited provider knowledge or engagement (Richardson and Paslakis 2021). Treatment access is further complicated by the fact that ED symptoms and body ideals can differ between genders (e.g., striving for muscularity vs. a thinness) (Forrest et al. 2019). Effective strategies to counteract such stigmatization in healthcare professionals remain unclear.

Previous studies suggest that combined approaches providing information and contact with those affected pose an effective anti‐stigma strategy, as in the Canadian Opening Minds Initiative (Rüsch et al. 2020; Stuart et al. 2014). Similar approaches have been explored in the context of EDs (Doley et al. 2017). In a German sample of women with an ED, general practitioners (GPs) were the main first point of diagnosis and referral for Anorexia nervosa (Neubauer et al. 2014), underscoring their role as a key target for ED anti‐stigma interventions. Although campaigns exist to inform about men with EDs (Bundesinstitut für Öffentliche Gesundheit (BIÖG), n.d.), there are no interventions that explicitly address GPs, despite their key role as “gatekeepers” in the German healthcare system (UK Parliament 2019). Thus, there is a need to act against the stigmatization of men with EDs among GPs in Germany.

Conceptually, iSMEsH targets ED‐related stigma as a multidimensional phenomenon that operates through cognitive, affective, and behavioral mechanisms. The intervention was based on a social‐cognitive, multi‐component model of stigma (Rüsch et al. 2005) and combines (i) education on male‐specific ED presentations to address knowledge deficits, (ii) contact with lived‐experience narratives to correct biased attitudes, and (iii) practical communication guidance to improve clinicians confidence in detection and referral (for more details, see Lehe et al. 2025).

### 1.1 Objectives

The primary aim of this randomized waitlist‐controlled study was to evaluate the efficacy of a lived experience co‐designed, online anti‐stigma training for German GPs in reducing stigmatizing attitudes toward men with EDs. Secondarily, we examined maintenance of effects over time. We hypothesized that, compared to a waitlist control condition, participation would increase knowledge of male‐specific ED presentations (cognitive facet), reduce stigmatizing attitudes (affective facet), and enhance self‐efficacy in identifying and managing ED symptoms (behavioral facet). A parallel study with medical students allowed comparison independent of systemic constraints shaping GPs' practice (e.g., workload, economic pressures).

## 2 Method

### 2.1 Trial Design, Recruitment, and Participants

We recruited GPs and medical students for a randomized waitlist‐controlled study between July 23, 2024 and May 10, 2025, through leaflets, personal referrals, and emails. GP addresses were obtained online and from a nationwide probabilistic registry sample (*n* = 10,000; please refer to Supplementary Text 1 for details). We included participants (i) aged ≥ 18 years, (ii) practicing as a GP or undergoing clinical training year, and (iii) treating men. As compensation, participants received a certificate of participation, access to Supporting Information, 1 CME point, and 25€ (GPs only).

An a priori power analysis (G*Power 3.1.9.7; Faul et al. 2007) for a 2 × 3 repeated‐measures ANOVA, assuming a power of 0.80, *α* = 0.05, moderate repeated measures correlations (*r* = 0.50), and perfect sphericity (ε = 1), suggested a minimum total *N* = 74 to detect a small‐to‐medium‐sized interaction effect (*f* = 0.15) within GP and student samples, respectively. Although anti‐stigma interventions show average medium effect sizes around *f* = 0.25 in between‐group comparisons (Doley et al. 2017), we planned for *f* = 0.15, considering that interaction effects are smaller in waitlist‐controlled comparisons (Sommet et al. 2023). We thus targeted *n* = 100 GPs and *n* = 100 students (i.e., *n* = 50 per condition). An additional re‐estimation of the sample size based on Sommet et al. (2023), assuming a “fully attenuated” interaction in a mixed‐factorial design, in which one group shows a medium‐sized effect (*d* = 0.50) over time, but not the other, resulted in a similar estimate of *N* = 99.

The study was funded by the Federal Ministry of Health (grant number ZMI5‐2523FSB212), had been prospectively registered (https://aspredicted.org/tzds‐h5yq.pdf), and reviewed and approved by the Ethics Committee of the Ruhr‐University Bochum's Medical Faculty at Campus East‐Westphalia (AZ 2023–1106; Nov. 7, 2023). All participants gave informed consent. We report all deviations from the study protocol (Lehe, Halbeisen, and Paslakis 2025). Materials are publicly available (Lehe, Sabel, Halbeisen, and Paslakis 2025).

### 2.2 Procedure

**FIGURE 1:**
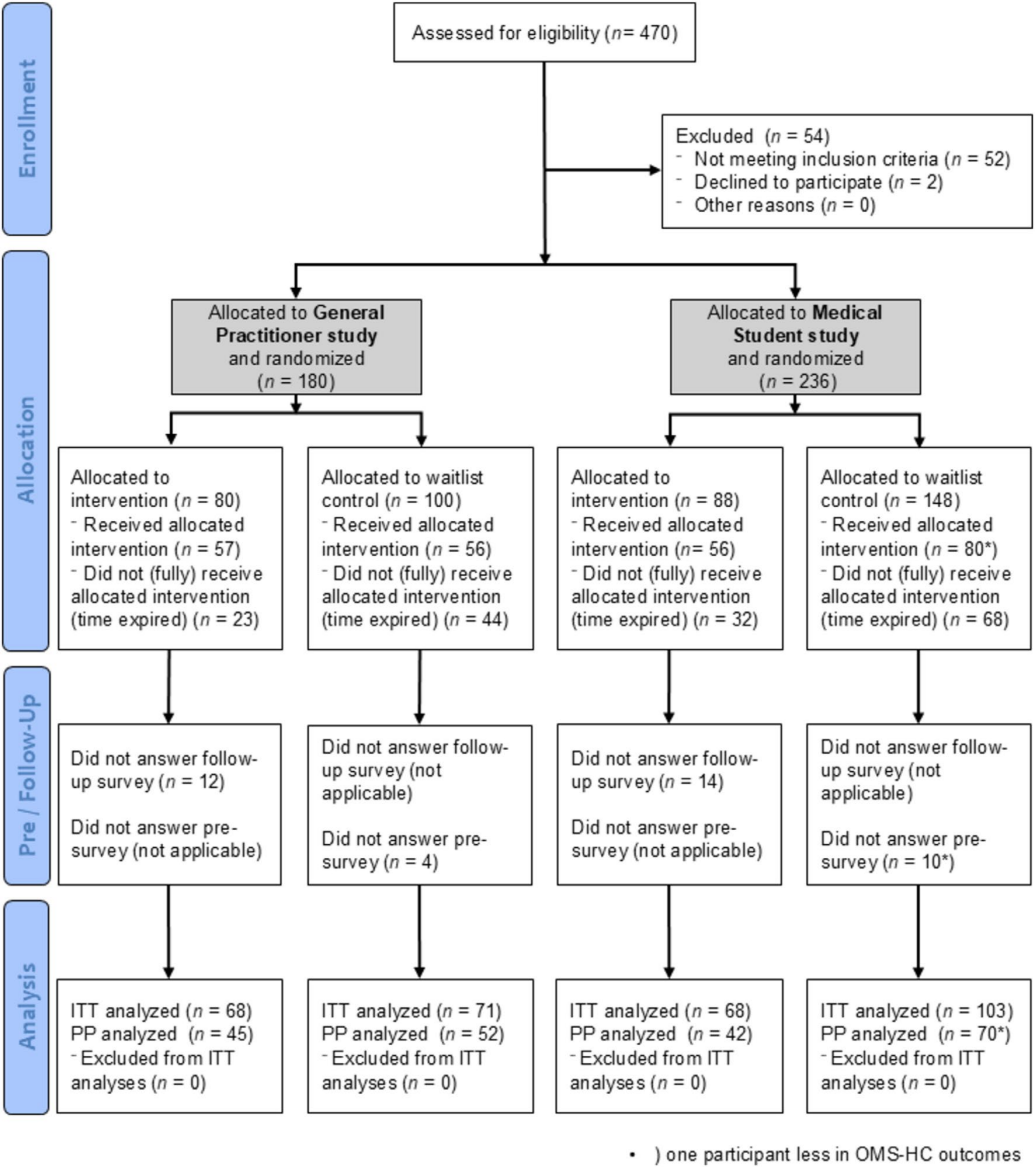
CONSORT flow chart for studies on general practitioners and on medical students. ITT, intention‐to‐treat; PP, per‐protocol; Time Expired, 14‐day intervention access period expired.

Eligible participants were randomly assigned in a 1:1 allocation ratio to either the intervention group (IG) or to a waitlist control group (CG; see Figure 1). The randomization sequence was generated using Microsoft Excel by the first author, and group allocation was implemented by a lab assistant, who was not involved in participant recruitment or assessment. The intervention was delivered via Moodle. IG participants received login credentials immediately after randomization for a 14‐day access period, followed by a follow‐up survey 14 days later. Waitlist CG participants received a pre‐survey link at randomization and access to the training after 14 days. Participants who did not complete the training modules, associated attention checks (“Did you watch the entire video?”; yes/no) following each video, and pre‐ and post‐assessments within 14 days were not considered per‐protocol completers; however, all participants were included in the ITT analysis irrespective of adherence or withdrawal. Pre‐participation (only for waitlist CG, administered 14 days prior to participation window) and follow‐up surveys (only for IG, 14 days after participation window) were handled as optional. All assessments were performed using Moodle's survey module and jsPsych (De Leeuw et al. 2023).

### 2.3 Intervention

iSMEsH is a video‐based, on‐demand online training co‐developed with men who have lived experience of an ED. We chose this format to ensure integrability into GPs' everyday work (Bayar et al. 2009; Sofyan and Meinel 2024a, 2024b). The six modules featured presentation slides, demonstrational dialogs, reflective exercises, and downloadable in‐depth resources (see Table 1). Each module could be completed in 5–10 min (total duration: 45–50 min), followed by interactive questions, ratings, and quizzes. Building on narratives of men with lived experience of an ED, the intervention employs medial contact‐based (i.e., narrative quotes/vignettes and exemplary dialogs) and educative anti‐stigma strategies.

**TABLE 1:** iSMEsH online training intervention modules and content.

Module	Title	Content
1	Introduction to eating disorders	Diagnostic criteria, clinical impression, and epidemiology of EDs in Boys and Men Etiological concepts Promoting sensitivity to diversity Implications of stigmatization as “second illness”
2	Symptoms and diagnostics	Clinical signs for suspected EDs on the psychological, social, and physical level Guiding questions for psychological and physical examination Standardized diagnostic instruments (questionnaires, interviews) Basic principles of somatic treatment (Compulsive) hospital referral and stepped care
3	Communication skills	Recommendations for inclusive and non‐discriminatory communication Directly addressing stigma with patients Basic principles and concepts of Motivational Interviewing Perspective‐taking exercise
4	Muscle dysmorphia	Diagnostic criteria, clinical impression, and epidemiology of muscle dysmorphia as a subtype of body dysmorphic disorder Role of physical exercise and substance abuse (e.g., anabolic steroids) Rootedness in masculinity ideals and male body image Reflexive exercise on own internalized gender roles and masculinity concepts
5	Therapeutical, social and other support services	Barriers to care for boys and men Role of self and public stigmatization and structural discrimination Recommendations for adequate care Introduction to medical, psychotherapeutic, social, and other support services
6	Role and support of caregivers	Inviting, inclusive and non‐discriminatory communication with caregivers Role of caregivers in the treatment process Active support for caregivers

Abbreviations: ED, eating disorder; iSMEsH, intervention against the stigmatization of men with eating disorders in primary healthcare.

### 2.4 Outcomes

The cognitive, affective, and behavioral facets of ED‐related stigma served as the primary outcomes and were assessed at three timepoints: immediately before, after, and 14 days post‐intervention (IGs) or, respectively, 14 days prior, immediately before, and after the intervention (waitlist CGs). Cognitive stigma was operationalized in terms of knowledge regarding men's EDs using ten single‐choice items, which were specifically developed for iSMEsH and received accreditation by a medical professional organization. We used the total sum score for all analyses, with higher values indicating greater knowledge. The affective facet of stigma was assessed using an adaptation of the validated German version of the Opening Minds Stigma Scale for Health Care Providers (OMS‐HC) (Modgill et al. 2014; Zuaboni et al. 2021). The scale comprises 15 items assessing healthcare professionals' stigma, providing an overall global scale with the three subscales. Wording was adapted to refer to men with EDs by replacing the original phrasing (“people/person with a mental illness”) with “men with an eating disorder”. All items were rated on a 5‐point Likert scale ranging from 1 (*strongly disagree*) to 5 (*strongly agree*). We used the global scale mean score for our analyses (GPs: 0.70 ≤ *α* ≤ 0.79, Students: 0.63 ≤ *α* ≤ 0.82, across timepoints). Finally, for the behavioral facet, we assessed perceived self‐efficacy in treating boys and men with EDs, using the sum of three adapted items from the German version of the General Self‐Efficacy Scale (GSE) (Schwarzer and Jerusalem 1995, 1999) answered on a 4‐point scale ranging from 1 (*not at all true*) to 4 (*exactly true*). The adapted scale demonstrated acceptable reliability (GPs: 0.76 ≤ *α* ≤ 0.86, Students: 0.72 ≤ *α* ≤ 0.85, across all timepoints). For more details, please refer to Supplementary Text 2.

### 2.5 Additional Assessments

For exploration, we assessed stigma‐related perceptions of EDs in men directly using a previously developed seven‐item scale (Lehe et al. 2024). However, we refrained from reporting these results given the scales' insufficient reliability in the present samples.

We assessed participant characteristics, including detailed demographics, professional background, attitudes toward mental health, personal experience with mental impairments, professional experience with EDs, and job (GPs) or study characteristics (students). These variables are summarized in Table 2, while job and study‐related details are shown in Table S4.

**TABLE 2:** Participant characteristics for general practitioner and medical student samples as randomized.

Variable	General practitioners			Medical students		
	Total (*n* = 130)	Intervention group (*n* = 68)	Control group (*n* = 62)	Total (*n* = 162)	Intervention group (*n* = 68)	Control group (*n* = 94)
	*M* ± SD/*n* (%)	*M* ± SD/*n* (%)	*M* ± SD/*n* (%)	*M* ± SD/*n* (%)	*M* ± SD/*n* (%)	*M* ± SD/*n* (%)
Age^a^	43.45 ± 10.94	44.22 ± 11.47	42.61 ± 10.38	26.94 ± 3.13	26.65 ± 3.36	27.15 ± 2.94
Gender^b^						
Women	87 (67.44)	42 (62.69)	45 (72.58)	127 (78.40)	49 (72.06)	78 (82.98)
Men	41 (31.78)	25 (37.31)	16 (25.81)	31 (19.14)	17 (25.00)	14 (14.89)
Diverse/other	1 (0.78)	0 (0.00)	1 (1.61)	3 (1.85)	1 (1.47)	2 (2.13)
Prefer not to answer/not available	0 (0.00)	0 (0.00)	0 (0.00)	1 (0.62)	1 (1.47)	0 (0.00)
Sexual orientation^c^						
Heterosexual	111 (85.48)	58 (85.29)	53 (85.48)	110 (67.90)	52 (76.47)	58 (61.70)
Gay/lesbian	7 (5.38)	5 (7.35)	2 (3.23)	8 (4.94)	5 (7.35)	3 (3.19)
Bisexual/other	11 (8.46)	4 (5.88)	7 (11.29)	41 (25.31)	10 (14.71)	31 (32.98)
Prefer not to answer/not available	1 (0.77)	1 (1.47)	0 (0.00)	3 (1.85)	1 (1.47)	2 (2.13)
German language proficiency^d^						
Native language	119 (94.44)	63 (96.92)	56 (91.80)	150 (93.75)	62 (92.54)	88 (94.62)
Fluent proficiency	7 (5.56)	2 (3.08)	5 (8.20)	10 (6.25)	5 (7.46)	5 (5.38)
Basic knowledge	0 (0.00)	0 (0.00)	0 (0.00)	0 (0.0)	0 (0.0)	0 (0.0)
Migration background^e^						
Yes	22 (17.74)	8 (12.50)	14 (23.33)	31 (19.50)	14 (21.21)	17 (18.28)
No	102 (82.26)	56 (87.50)	46 (76.67)	128 (80.50)	52 (78.79)	76 (81.72)
Marital status^f^						
Single	29 (23.20)	15 (23.08)	14 (23.33)	152 (95.60)	64 (95.52)	88 (95.65)
Married/registered partnership	83 (66.40)	44 (67.69)	39 (65.00)	7 (4.40)	3 (4.48)	4 (4.35)
Widowed	1 (0.80)	0 (0.00)	1 (1.67)	0 (0.00)	0 (0.00)	0 (0.00)
Divorced	12 (9.60)	6 (9.23)	6 (10.00)	0 (0.00)	0 (0.00)	0 (0.00)
Living with disability^g^						
Yes	5 (4.00)	3 (4.69)	2 (3.28)	10 (6.33)	4 (6.15)	6 (6.45)
No	120 (96.00)	61 (95.31)	59 (96.72)	148 (93.67)	61 (93.85)	87 (93.55)
Racial minority status^h^						
Yes	5 (3.97)	2 (3.08)	3 (4.92)	14 (8.81)	6 (8.96)	8 (8.70)
No	120 (95.24)	62 (95.38)	58 (95.08)	143 (89.94)	59 (88.06)	84 (91.30)
Don't know	1 (0.79)	1 (1.54)	0 (0.00)	2 (1.26)	2 (2.99)	0 (0.00)
Personal history of an ED^i^						
Yes	8 (6.15)	7 (10.29)	1 (1.61)	15 (9.38)	6 (8.96)	9 (9.68)
No	122 (93.85)	61 (89.71)	61 (98.39)	143 (89.38)	61 (91.04)	82 (88.17)
Don't know	0 (0.00)	0 (0.00)	0 (0.00)	2 (1.25)	0 (0.00)	2 (2.15)
Personal history of ED treatment^j^						
Yes	6 (4.62)	5 (7.35)	1 (1.61)	13 (8.02)	5 (7.35)	8 (8.51)
No	35 (26.92)	19 (27.94)	16 (25.81)	46 (28.40)	23 (33.82)	23 (24.47)
Not applicable	27 (20.77)	12 (17.65)	15 (24.19)	55 (33.95)	16 (23.53)	39 (41.49)
Not answered	62 (47.69)	32 (47.06)	30 (48.39)	48 (29.63)	24 (35.29)	24 (25.53)
History of loved one with an ED^k^						
Yes	31 (23.85)	18 (26.47)	13 (20.97)	74 (45.96)	28 (41.18)	46 (49.46)
No	95 (73.08)	48 (70.59)	47 (75.81)	79 (49.07)	37 (54.41)	42 (45.16)
Don't know	4 (3.08)	2 (2.94)	2 (3.23)	8 (4.97)	3 (4.41)	5 (5.38)
Restraint in initial assignment of psychiatric diagnoses^l^	3.02 ± 1.19	3.12 ± 1.29	2.92 ± 1.08	2.56 ± 1.31	2.51 ± 1.33	2.60 ± 1.31
Refrains from mental health treatment referral^m^	4.65 ± 0.66	4.53 ± 0.80	4.77 ± 0.42	4.20 ± 1.02	4.15 ± 1.00	4.23 ± 1.04
Previously has offered eating disorder treatment^n^						
Yes	105 (80.77)	55 (80.88)	50 (80.65)	60 (37.04)	22 (32.35)	38 (40.43)
No	20 (15.38)	10 (14.71)	10 (16.13)	86 (53.09)	40 (58.82)	46 (48.94)
Don't know	5 (3.85)	3 (4.41)	2 (3.23)	16 (9.88)	6 (8.82)	10 (10.64)

*Note:* Unless otherwise indicated by superscript letters, the response options correspond to the row labels in the table. For further participant details, please refer to Table S4.

^a^ Item: How old are you?

^b^ Item: Which gender do you identify with?

^c^ Item: Which genders do you feel romantically/sexually attracted to?

^d^ Item: What is your level of German language proficiency?

^e^ Item: Do you have a migrant background, that is, were you or at least one of your parents born without German citizenship?

^f^ Item: What is your marital status?

^g^ Item: Do you live with a disability?

^h^ Item: Do you identify yourself as belonging to an ethnic minority or racialized group?

^i^ Item: Have you ever been diagnosed with an eating disorder?

^j^ Item: If so, are you currently receiving or have you previously received psychotherapeutic and/or medical treatment for this condition?

^k^ Item: Do you have a close friend or family member who has been diagnosed with an eating disorder?

^l^ Item: I avoid assigning a confirmed F‐diagnosis to patients for the first time. Response: Rated on a 5‐point Likert scale from 1 = *fully agree* to 5 = *fully disagree*.

^m^ Item: I avoid referring patients to psychiatric, psychosomatic, or psychotherapeutic treatment. Response: Rated on a 5‐point Likert scale from 1 = *fully agree* to 5 = *fully disagree*.

^n^ Item: Have you ever treated someone with an eating disorder?

Feasibility and other implementation‐related outcomes will be reported as a separate article.

### 2.6 Statistical Analysis

We conducted two sets of analyses for each primary outcome separately for the GP and student samples. First, we performed a 2 (group: IG vs. waitlist CG) × 3 (time: pre vs. post vs. follow‐up) *intention‐to‐treat* (ITT) analysis using linear mixed models (LMMs) with participant ID included as random intercept to account for repeated measures on all available data. Second, we conducted a corresponding 2 × 3 *per‐protocol* (PP) mixed ANOVA, with case‐wise exclusion for missing data (i.e., including only participants with complete datasets). The ITT analyses constituted the primary analyses and are reported in the Results section, whereas the PP analyses served as sensitivity analyses; their methodology and results are presented in Supplementary Text 3 and Tables S1 and S2. ITT analyses were conducted with *lmerTest* version 3.1‐3 (Kuznetsova et al. 2017). Models included by‐participant random intercepts and were estimated using restricted maximum likelihood estimation (REML). Main and interaction effects were evaluated using Type‐III Wald χ^2^ tests for the fixed effects and estimated confidence intervals using a bootstrapping procedure with 1000 iterations. We used Q‐Q plots to check for non‐linearity and heteroscedasticity. Analyses of missingness conducted using *missr* version 1.0.1 (Hellen 2025) supported the assumption that the missing‐data mechanism in the present dataset was consistent with at least missing at random. Given the robustness of LMMs against other violations of distributional assumptions (Schielzeth et al. 2020), we did not conduct further assumption checks. Post hoc tests based on EMMs were then conducted to examine the predicted interaction patterns; pre‐to‐post changes among intervention participants were examined with paired‐samples *t*‐tests.

All data were analyzed using *R* version 4.5.1 (R Core Team 2025). We report means (*M*) and standard deviations (SD) for continuous variables, and frequencies (*n*) and percentages (%) for categorical variables. Internal consistencies were evaluated as Cronbach's *α* and interpreted following George and Mallery (2016). Between‐group comparisons used Student's *t*‐tests (Welch's *t*‐tests if variances were unequal) and chi‐square tests for categorical data. Effect sizes are reported as Cohen's *d* for *t*‐tests and LMM interaction effects, and partial η^2^ for ANOVAs. Statistical significance was set at *p* ≤ 0.05. Visualizations were generated using *ggplot2* version 3.5.2 (Wickham 2016). Data quality was assessed using attention check items following each module and all cases were included in the analyses.

## 3 Results

### 3.1 Sample Characteristics and Adherence

Participants' demographics, mental health attitudes, mental health history, and professional experience with patients with EDs are summarized in Table 2. Within the GP population, the CG showed less restraint in referring patients to mental health treatment compared to the IG. Additionally, the IG and CGs of medical students differed in sexual orientation. There were no other group differences in sample characteristics. Overall, 42.26% of IG participants and 35.08% of waitlist CG participants completed all six modules (*M*_IG_ = 3.77, SD_IG_ = 2.54, *M*_waitlist CG_ = 3.11, SD_waitlist CG_ = 2.70). For detailed adherence data, please refer to Table S3.

### 3.2 General Practitioners

**FIGURE 2:**
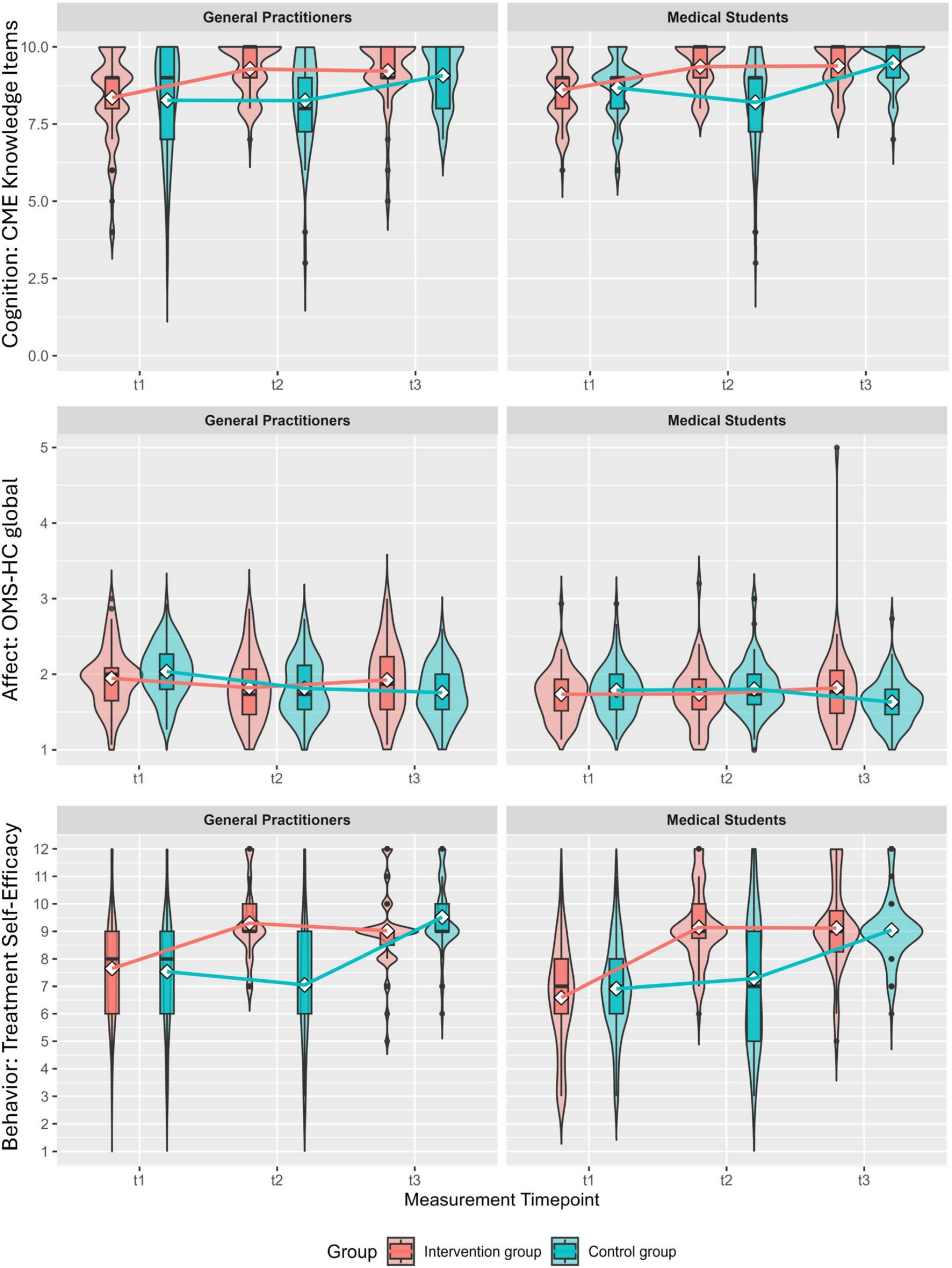
Anti‐stigma efficacy for all facets of stigma by group for both general practitioner and medical student samples.

As expected, the 2 (group) × 3 (time) LMM analyses demonstrated significant group‐by‐time interactions across outcomes (see Table 3 for fixed effects results, Figure 2 for visualizations, Table 4 for descriptives, and Table S5 for coefficient‐level statistics), all *X* ^2^s ≥ 7.69, all *p*s ≤ 0.021, all η_p_^2^s ≥ 0.03.

**TABLE 3:** Linear mixed‐model fixed effects for stigma facet outcomes by population (intention‐to‐treat analyses).

Sample	DV	Group	Time	Group × time
GPs	Cognition	*X* ^2^ (1) = 0.15, *p* = 0.695, η_p_^2^ = 0.08	*X* ^2^ (2) = 19.98, *p* < 0.001***, η_p_^2^ = 0.10	*X* ^2^ (2) = 10.13, *p* = 0.006**, η_p_^2^ = 0.04
	Affect	*X* ^2^ (1) = 1.82, *p* = 0.177, η_p_^2^ = 0.00	*X* ^2^ (2) = 3.78, *p* = 0.151, η_p_^2^ = 0.06	*X* ^2^ (2) = 7.69, *p* = 0.021*, η_p_^2^ = 0.03
	Behavior	*X* ^2^ (1) = 0.13, *p* = 0.723, η_p_^2^ = 0.07	*X* ^2^ (2) = 34.25, *p* < 0.001***, η_p_^2^ = 0.19	*X* ^2^ (2) = 43.11, *p* < 0.001***, η_p_^2^ = 0.15
Students	Cognition	*X* ^2^ (1) = 0.14, *p* = 0.708, η_p_^2^ = 0.06	*X* ^2^ (2) = 22.55, *p* < 0.001***, η_p_^2^ = 0.13	*X* ^2^ (2) = 34.14, *p* < 0.001***, η_p_^2^ = 0.10
	Affect	*X* ^2^ (1) = 0.79, *p* = 0.374, η_p_^2^ = 0.00	*X* ^2^ (2) = 1.72, *p* = 0.424, η_p_^2^ = 0.00	*X* ^2^ (2) = 9.79, *p* = 0.007**, η_p_^2^ = 0.03
	Behavior	*X* ^2^ (1) = 1.47, *p* = 0.225, η_p_^2^ = 0.05	*X* ^2^ (2) = 92.04, *p* < 0.001***, η_p_^2^ = 0.31	*X* ^2^ (2) = 35.26, *p* < 0.001***, η_p_^2^ = 0.11

*Note:* Results are from linear mixed‐effects models with random intercepts for participants. We report Wald χ^2^ tests (Type III) for fixed effects as *X* ^2^ (df). For full coefficient term statistics of the linear mixed‐effects models, please refer to Table S5. *p < 0.05, **p < 0.01, ***p < 0.001.

Abbreviations: Affect, opening minds stigma scale for healthcare providers (OMS‐HC); Behavior, general self‐efficacy scale (GSE); Cognition, continuing medical education (CME) knowledge items; DV, dependent variable; GPs, general practitioners; Students, medical students.

**TABLE 4:** Descriptive statistics by population, timepoint, and group for stigma facet outcomes (intention‐to‐treat analyses).

Sample	DV	Timepoint	Total		Intervention group		Control group	
			*N* of completers	M ± SD	*N* of completers	M ± SD	*N* of completers	M ± SD
GPs	Cognition	T1	139	8.31 ± 1.54	68	8.35 ± 1.41_a_	71	8.27 ± 1.66_a_
		T2	119	8.75 ± 1.31	57	9.28 ± 0.84_a_	62	8.26 ± 1.47_b_
		T3	103	9.14 ± 1.01	47	9.21 ± 1.06_a_	56	9.07 ± 0.97_a_
	Affect	T1	139	1.99 ± 0.38	68	1.95 ± 0.40_a_	71	2.04 ± 0.35_a_
		T2	119	1.82 ± 0.40	57	1.82 ± 0.43_a_	62	1.81 ± 0.38_a_
		T3	103	1.83 ± 0.41	47	1.92 ± 0.47_a_	56	1.76 ± 0.35_b_
	Behavior	T1	139	7.59 ± 1.91	68	7.65 ± 1.81_a_	71	7.54 ± 2.02_a_
		T2	119	8.13 ± 2.15	57	9.30 ± 1.35_a_	62	7.05 ± 2.19_b_
		T3	103	9.29 ± 1.35	47	9.02 ± 1.39_a_	56	9.52 ± 1.28_a_
Students	Cognition	T1	171	8.64 ± 1.00	68	8.60 ± 1.02_a_	103	8.67 ± 0.99_a_
		T2	150	8.63 ± 1.39	56	9.36 ± 0.77_a_	94	8.20 ± 1.49_b_
		T3	122	9.45 ± 0.72	42	9.38 ± 0.73_a_	80	9.49 ± 0.71_a_
	Affect	T1	171	1.77 ± 0.33	68	1.73 ± 0.33_a_	103	1.79 ± 0.33_a_
		T2	150	1.78 ± 0.36	56	1.74 ± 0.38_a_	94	1.80 ± 0.34_a_
		T3	122	1.70 ± 0.44	42	1.82 ± 0.62_a_	80	1.63 ± 0.30_b_
	Behavior	T1	171	6.78 ± 1.76	68	6.59 ± 1.90_a_	103	6.91 ± 1.66_a_
		T2	150	7.97 ± 2.20	56	9.14 ± 1.35_a_	94	7.28 ± 2.32_b_
		T3	122	9.07 ± 1.31	42	9.12 ± 1.60_a_	80	9.05 ± 1.15_a_

*Note:* Means with different subscript letters are statistically different at *p* < 0.05 of a Bonfferoni‐adjusted pairwise comparison Student's *t*‐test.

Abbreviations: Affect, opening minds stigma scale for healthcare providers (OMS‐HC); Behavior, general self‐efficacy scale (GSE); Cognition, continuing medical education (CME) knowledge items; DV, dependent variable; GPs, general practitioners; Students, medical students.

The GPs in the IG demonstrated increased knowledge (i.e., reduction of cognitive stigma) from baseline to post‐intervention, *p*_T1–T2_ < 0.001, *d*_T1–T2_ = −0,72, 95% CI (−1.08, −0.37), which remained stable at follow up, *p*_T2–T3_ > 0.999, *d*_T2–T3_ = 0.06, 95% CI (−0.33, 0.45). In contrast, GPs in the waitlist CG showed no change between baseline and pre‐intervention, *p*_T1–T2_ > 0.999, *d*_T1–T2_ = 0.01, 95% CI (−0.34, 0.35), but exhibited a significant knowledge gain following the intervention, *p*_T2–T3_ = 0.002, *d*_T2–T3_ = −0.64, 95% CI (−1.00, −0.27). From a between‐group perspective, GPs in IG and CG did not differ at baseline or at study end, *ps* ≥ 0.598, *d*_IG–CG_ = 0.07, 95% CI (−0.27, 0.41) and *d*_IG–CG_ = 0.11, 95% CI (−0.29, 0.50), respectively, whereas the IG showed the expected advantage over the waitlist CG immediately after intervention completion, when the CG had not yet received the training, *p* < 0.001, *d*_IG–CG_ = 0.80, 95% CI (0.43, 1.17).

Regarding affective stigma, the IG did not show a significant reduction in stigmatizing attitudes from baseline to post‐intervention, *p*_T1–T2_ = 0.212, *d*_T1–T2_ = 0.33, 95% CI (−0.03, 0.69), or from post‐intervention to follow‐up, *p*_T2–T3_ = 0.416, *d*_T2–T3_ = −0.30, 95% CI (−0.69, 0.10). In contrast, the waitlist CG exhibited a significant decrease in stigmatizing attitudes during the waiting period, *p*_T1–T2_ = 0.002, *d*_T1–T2_ = 0.62, 95% CI (0.27, 0.97), which persisted through follow‐up, *p*_T1–T3_ < 0.001, *d*_T1–T3_ = 0.77, 95% CI (0.41, 1.13), with no further reduction during intervention participation, *p*_T2–T3_ > 0.999, *d*_T2–T3_ = 0.15, 95% CI (−0.22, 0.51). The two GP groups did not differ significantly at baseline or at T2, *ps* ≥ 0.178, *d*_IG–CG_ = −0.26, 95% CI (−0.63, 0.12) and *d*_IG–CG_ = 0.04, 95% CI (−0.37, 0.44), respectively, but differed significantly at study end, *p* = 0.030, *d*_IG–CG_ = 0.48, 95% CI (0.05, 0.92).

Finally, regarding treatment self‐efficacy, the data showed the full expected pattern. GPs in the IG demonstrated increased self‐efficacy from baseline to post‐intervention, *p*_T1–T2_ < 0.001, *d*_T1–T2_ = −0.98, 95% CI (−1.34, −0.62), which was maintained at follow‐up, *p*_T2–T3_ > 0.999, *d*_T2–T3_ = 0.16, 95% CI (−0.23, 0.55). In contrast, participants in the waitlist CG did not report self‐efficacy gains during the waiting period, *p*_T1–T2_ = 0.258, *d*_T1–T2_ = 0.30, 95% CI (−0.04, 0.65), but showed a significant increase over the intervention, *p*_T2–T3_ < 0.001, *d*_T2–T3_ = −1.49, 95% CI (−1.87, −1.11). Accordingly, the groups differed at T2, after the IG had received the intervention, *p* < 0.001, *d*_IG–CG_ = 1.35, 95% CI (0.96, 1.74), but not at baseline or at study end, *ps* ≥ 0.140, *d*_IG–CG_ = 0.06, 95% CI (−0.29, 0.41) and *d*_IG–CG_ = −0.31, 95% CI (−0.72, 0.10).

These findings were largely consistent with the PP sensitivity analyses (see Supplementary Text 3 and Tables S1 and S2).

### 3.3 Medical Students

In the student sample, the 2 (group) × 3 (time) mixed LMM analyses confirmed the anticipated group‐by‐time interactions across outcomes (see Table 3 for fixed effects results, Figure 2 for visualizations, Table 4 for descriptives, and Table S5 for coefficient‐level statistics), all *X* ^2^s ≥ 9.79, all *p*s ≤ 0.007, all *η*_*p*_^2^ *s* ≥ 0.03.

Students in the IG showed a significant increase in knowledge from baseline to post‐intervention, *p*_T1–T2_ < 0.001, *d*_T1–T2_ = −0.74, 95% CI (−1.10, −0.38), which remained stable at follow‐up, *p*_T2–T3_ > 0.999, *d*_T2–T3_ = −0.02, 95% CI (−0.43, 0.38). Contrary to our hypotheses, students in the waitlist CG initially exhibited a significant decrease in knowledge during the waiting period, *p*_T1–T2_ = 0.005, *d*_T1–T2_ = 0.46, 95% CI (0.17, 0.74), followed by a significant knowledge gain after intervention completion, *p*_T2–T3_ < 0.001, *d*_T2–T3_ = −1.26, 95% CI (−1.57, −0.95). There were no between‐group differences at baseline or at study end, *p*s ≥ 0.596, *d*_IG–CG_ = −0.06, 95% CI (−0.38, 0.26) and *d*_IG–CG_ = −0.10, 95% CI (−0.49, 0.28), whereas the IG showed a temporary advantage over the waitlist CG immediately after training completion, *p* < 0.001, *d*_IG–CG_ = 1.14, 95% CI (0.79, 1.49).

Regarding affective stigma, the IG did not show a significant reduction in stigmatizing attitudes either from baseline to post‐intervention, *p*_T1–T2_ > 0.999, *d*_T1–T2_ = −0.01, 95% CI (−0.37, 0.35), or from post‐intervention to follow‐up, *p*_T2‐T3_ = 0.785, *d*_T2–T3_ = −0.23, 95% CI (−0.64, 0.17). In contrast, the waitlist CG showed no change during the waiting period, *p*_T1–T2_ > 0.999, *d*_T1–T2_ = −0.07, 95% CI (−0.35, 0.22), but exhibited a significant decrease over the intervention phase, *p*_T2–T3_ = 0.004, *d*_T2–T3_ = 0.50, 95% CI (0.20, 0.81). The two groups did not differ significantly at baseline or at T2, *ps* ≥ 0.257, *d*_IG–CG_ = −0.15, 95% CI (−0.49, 0.19) and *d*_IG–CG_ = −0.21, 95% CI (−0.57, 0.15), but differed significantly at study end, *p* = 0.012, *d*_IG–CG_ = 0.53, 95% CI (0.11, 0.94), indicating that only the CG demonstrated the expected reduction in affective stigma.

With respect to treatment self‐efficacy, the students' data showed the fully anticipated pattern. Participants in the IG demonstrated increased self‐efficacy from baseline to post‐intervention, *p*_T1–T2_ < 0.001, *d*_T1–T2_ = −1.53, 95% CI (−1.90, −1.16), with gains maintained at follow‐up, *p*_T2–T3_ > 0.999, *d*_T2–T3_ = 0.01, 95% CI (−0.39, 0.42). In contrast, participants in the waitlist CG did not report significant changes during the waiting period, *p*_T1–T2_ = 0.449, *d*_T1–T2_ = −0.21, 95% CI (−0.49, 0.08), but exhibited significant gains over the intervention, *p*_T2–T3_ < 0.001, *d*_T2–T3_ = −1.05, 95% CI (−1.36, −0.74). Accordingly, the groups did not differ at baseline or at study end, *ps* ≥ 0.226, *d*_IG–CG_ = −0.20, 95% CI (−0.52, 0.12) and *d*_IG–CG_ = 0.06, 95% CI (−0.33, 0.45), but the IG reported higher self‐efficacy than the CG immediately after completing the intervention, *p* < 0.001, *d*_IG–CG_ = 1.12, 95% CI (0.77, 1.48).

These findings were largely consistent with those obtained in the PP sensitivity analyses (see Supplementary Text 3 and Tables S1 and S2), underscoring the robustness of the observed effects.

### 3.4 Exploratory Analyses

Because the interventions and measures were identical for GPs and medical students, we additionally analyzed the combined sample using the aforementioned LMMs to examine potential population‐based moderation effects of group × time interactions. Including population as a factor improved model fit in two of the three models (i.e., the affective and behavioral facets of stigma). However, there was no evidence for population‐based moderation (all *ps* > 0.166), and analyses are therefore not reported further. However, the study was not powered to detect three‐way interactions; accordingly, these null findings should be interpreted with caution.

## 4 Discussion

EDs in men are frequently underdiagnosed, contributing to a persistent gender treatment gap (Halbeisen et al. 2024). Beyond gender differences in symptom presentation (Brown and Keel 2023; Ruffo et al. 2025), stigma—such as viewing EDs as “women's disorders”—can pose a barrier to men's help‐seeking and hinder recognition and care provision (Bomben et al. 2022; Lehe, Halbeisen, Juergensen, et al. 2025; Mycock et al. 2025). Thus, such stigma represents a biased social construction of EDs rooted in cultural understandings of gender (Fixsen 2024; Räisänen and Hunt 2014), shapes how affected individuals perceive themselves (Griffiths et al. 2015) and influences healthcare professionals' attitudes (Richardson and Paslakis 2021). Therefore, targeted interventions addressing ED stigma in men are needed to improve timely detection and referral.

This randomized waitlist‐controlled study evaluated the efficacy of an online, lived‐experience co‐designed anti‐stigma intervention for German GPs and medical students. The intervention aimed to reduce stigmatizing attitudes toward men with EDs and enhance knowledge and treatment self‐efficacy. The intervention was effective in reducing cognitive (i.e., increasing knowledge) and behavioral stigma (i.e., enhancing treatment self‐efficacy) in both GPs and students, whereas effects on affective stigma were less consistent.

Our results align with prior research showing that combined education and contact‐based approaches can reduce healthcare stigma. Comparable online and in‐person ED‐focused anti‐stigma interventions—although often targeting “classical” ED presentations and using less rigorous designs—also reported improvements in provider knowledge or self‐efficacy (Bronlow et al. 2015; Gurney and Halmi 2001). The consistency between ITT and PP analyses further supports the robustness and practical relevance of the observed effects.

However, the less consistent effects on affective stigma may reflect the difficulty of countering deeply ingrained attitudes and prejudices within a short timeframe. While descriptive trends were evident in some analyses, only the student waiting CG showed a significant reduction in affective stigma during the waiting period in the ITT analysis, and this reduction remained stable following subsequent intervention participation. This finding may relate to differences in intervention scope and outcome definitions. Bronlow et al. (2015) observed reductions of affective stigma after a substantially longer online training of 3.5 h with a 6‐month participation window using non‐validated measures. Gurney and Halmi (2001) also reported changes in attitudes over an in‐person training; however, their definition of attitudes was based on participants' perceived ability to intervene, which reflects the behavioral facet of stigma in our understanding, for which we also found a significant increase. Several factors may account for the non‐significant affective stigma reduction in our study. First, the brief intervention duration and the 14‐day completion window may have limited exposure and led to participant drop‐out (IG: 35.1%, CG: 46.0%). Second, the short follow‐up interval might not have been sufficient, as affective changes are typically slower to manifest than cognitive or behavioral ones. Third, the OMS‐HC measure may not have been sufficiently sensitive to detect change. Finally, as affective stigma may be rooted in longstanding cultural narratives and emotional reactions, our brief knowledge‐focused intervention, including indirect contact through video, may per se not have been adequate to shift affective responses. Thus, to address affective stigma, interventions complemented by personal interactions or reflexive exercises that foster empathy and facilitate deeper personal engagement may be more appropriate. Notably, the reduction observed during the waiting period in the student CG could reflect a waiting effect rather than a true intervention impact. Overall, the intervention yielded small‐to‐moderate effect sizes comparable to those reported in previous anti‐stigma interventions (Doley et al. 2017).

Exploratory analyses on primary outcomes revealed no population‐specific differences in intervention effects; given limited power, this finding may serve as a preliminary indication of the intervention's applicability across clinical and educational contexts.

The low internal consistency of the secondary outcome stigma measure—derived from affected men's narratives—may indicate that it primarily captures self‐stigma, whereas healthcare professionals may be more influenced by public stigma.

### 4.1 Strengths and Limitations

This study has several strengths, including the randomized waitlist‐controlled design, substantial sample size, and inclusion of both GPs and medical students, which allows to compare effects with and without systemic constraints of the healthcare system (e.g., workload). Furthermore, participatory development with men with lived‐experience and multidimensional stigma assessment grounded in established frameworks (Link and Phelan 2001; Rüsch et al. 2005) support conceptual and ecological validity. It is, to the best of our knowledge, the first German intervention specifically targeting EDs in men.

However, there are limitations to consider. First, the study relied on self‐reported measures, which may be influenced by social desirability bias. In addition, the instruments were adapted to fit the study context, as no established measures were available that fully captured the constructs of interest in this setting. While steps were taken to support validity and reliability, these adaptations cannot fully replace a comprehensive psychometric scale development. A related limitation is that, due to the challenges of assessing objective markers of behavior change in an online study, the behavioral component of stigma was operationalized using treatment self‐efficacy as a behavior‐relevant proxy rather than a direct measure of actual clinical behavior. Future research should develop and incorporate more direct behavioral indicators of stigmatization while accounting for social desirability bias. Second, the waitlist condition might have led to slight changes due to anticipation effects, as observed in other interventions (Steinert et al. 2017). Nonetheless, consistent effects across IGs and CGs, different populations, and both ITT and PP analyses support the robustness of the findings. Third, repeated assessments could have induced minor learning effects. Finally, generalizability may be limited to the German healthcare context, although our results align with findings from other countries.

Future research should replicate and extend these findings to other contexts, develop and employ more precise and change‐sensitive measures of ED stigma, aim for larger samples to ensure adequate power for the detection of three‐way interactions to inform population‐tailored interventions, and assess long‐term effects through extended follow‐up periods to evaluate the sustainability of intervention outcomes.

### 4.2 Implications for Clinical Practice and Policy

The iSMEsH intervention effectively reduced specific facets of stigma, highlighting its potential to improve early detection and treatment of EDs in men, support timely specialist care, and help close the gender treatment gap, thereby supporting men's human right to equal healthcare access. The findings can inform the development and evaluation of future anti‐stigma interventions in healthcare contexts. All materials and resources are freely available (Lehe, Sabel, Halbeisen, and Paslakis 2025). The intervention also aligns with national policy efforts in Germany, including the Federal Institute for Public Health's (BIÖG) campaign on EDs and the national suicide prevention strategy. Finally, the intervention illustrates how gender‐sensitive care can enhance health outcomes for both women and men.

## 5 Conclusions

In conclusion, this study provides evidence for the efficacy of a lived experience co‐designed, online anti‐stigma training targeting stigma of EDs in men by increasing knowledge and self‐efficacy among GPs and medical students. While the effects on affective stigma need to be assessed in more detail in future research, the intervention shows promise as a tool for enhancing the early detection and treatment of EDs in men.

## Author Contributions

**Martin S. Lehe:** conceptualization, methodology, software, validation, formal analysis, investigation, data curation, writing – original draft, writing – review and editing, visualization, project administration. **Georg Halbeisen:** conceptualization, methodology, software, writing – review and editing, project administration, funding acquisition. **Sabine Steins‐Loeber:** resources, writing – review and editing. **Georgios Paslakis:** conceptualization, resources, writing – review and editing, supervision, funding acquisition.

## Funding

This project was funded by the German Federal Ministry of Health (Bundesministerium für Gesundheit) based on a resolution of the German Bundestag, grant number ZMI5‐2523FSB212, awarded to Georgios Paslakis.

## Ethics Statement

The study obtained ethical approval by the Ethics Committee of the Ruhr‐University Bochum's Medical Faculty at Campus East‐Westphalia (AZ 2023–1106) on November 7, 2023, and was prospectively registered on July 1, 2024 (#181,415; https://aspredicted.org/tzds‐h5yq.pdf). A study protocol is published under Lehe, Halbeisen, and Paslakis (2025).

## Consent

All authors consented to publication. Details may be found in the attached document.

## Conflicts of Interest

The authors declare no conflicts of interest.

## Supporting information


**Data S1:** eat70080‐sup‐0001‐Supinfo.docx.

## Data Availability

The data that support the findings of this study are available from the corresponding author upon reasonable request.

## References

[eat70080-bib-0001] Bayar, M. R. , B. C. Poyraz , C. Aksoy‐Poyraz , and M. K. Arikan . 2009. “Reducing Mental Illness Stigma in Mental Health Professionals Using a Web‐Based Approach.” Israel Journal of Psychiatry and Related Sciences 46, no. 3: 226–230. 20039525

[eat70080-bib-0002] Bomben, R. , N. Robertson , and S. Allan . 2022. “Barriers to Help‐Seeking for Eating Disorders in Men: A Mixed‐Methods Systematic Review.” Psychology of Men & Masculinities 23, no. 2: 183–196. 10.1037/men0000382.

[eat70080-bib-0003] Brelet, L. , V. Flaudias , M. Désert , S. Guillaume , P.‐M. Llorca , and Y. Boirie . 2021. “Stigmatization Toward People With Anorexia Nervosa, Bulimia Nervosa, and Binge Eating Disorder: A Scoping Review.” Nutrients 13, no. 8: 8. 10.3390/nu13082834. PMC8400545 34444994

[eat70080-bib-0004] Bronlow, R. S. , S. Maguire , A. O'Dell , C. Dias‐da‐Costa , S. Touyz , and J. Russell . 2015. “Evaluation of an Online Training Program in Eating Disorders for Health Professionals in Australia.” Journal of Eating Disorders 3, no. 37. 10.1186/s40337-015-0078-7. PMC4636783 26550477

[eat70080-bib-0005] Brown, T. A. , and P. K. Keel . 2023. “Eating Disorders in Boys and Men.” Annual Review of Clinical Psychology 19, no. 1: 177–205. 10.1146/annurev-clinpsy-080921-074125. 36737595

[eat70080-bib-0006] Bundesinstitut für Öffentliche Gesundheit (BIÖG) . n.d. “Essstörungen bei Jungen und Männern. BZgA Essstörungen.” https://www.bzga‐essstoerungen.de/habe‐ich‐eine‐essstoerung/essstoerungen‐bei‐jungen‐und‐maennern/.

[eat70080-bib-0007] De Leeuw, J. R. , R. A. Gilbert , and B. Luchterhandt . 2023. “jsPsych: Enabling an Open‐Source Collaborative Ecosystem of Behavioral Experiments.” Journal of Open Source Software 8, no. 85: 5351. 10.21105/joss.05351.

[eat70080-bib-0008] Doley, J. R. , L. M. Hart , A. A. Stukas , K. Petrovic , A. Bouguettaya , and S. J. Paxton . 2017. “Interventions to Reduce the Stigma of Eating Disorders: A Systematic Review and Meta‐Analysis.” International Journal of Eating Disorders 50, no. 3: 210–230. 10.1002/eat.22691. 28230911

[eat70080-bib-0009] Faul, F. , E. Erdfelder , A.‐G. Lang , and A. Buchner . 2007. “G*Power 3: A Flexible Statistical Power Analysis Program for the Social, Behavioral, and Biomedical Sciences.” Behavior Research Methods 39, no. 2: 175–191. 10.3758/BF03193146. 17695343

[eat70080-bib-0010] Ferrari, A. J. , D. F. Santomauro , A. M. M. Herrera , et al. 2022. “Global, Regional, and National Burden of 12 Mental Disorders in 204 Countries and Territories, 1990–2019: A Systematic Analysis for the Global Burden of Disease Study 2019.” Lancet Psychiatry 9, no. 2: 137–150. 10.1016/S2215-0366(21)00395-3. 35026139 PMC8776563

[eat70080-bib-0011] Fixsen, A. 2024. The Construction of Eating Disorders: Psychiatry, Politics and Cultural Representations of Disordered Eating. Springer Nature Switzerland. 10.1007/978-3-031-70318-8.

[eat70080-bib-0012] Flores, L. E. , R. Muir , I. Weeks , H. Burton Murray , and J. K. Silver . 2022. “Analysis of Age, Race, Ethnicity, and Sex of Participants in Clinical Trials Focused on Eating Disorders.” JAMA Network Open 5, no. 2: e220051. 10.1001/jamanetworkopen.2022.0051. 35188557 PMC8861843

[eat70080-bib-0013] Forrest, L. N. , N. M. Perkins , J. M. Lavender , and A. R. Smith . 2019. “Using Network Analysis to Identify Central Eating Disorder Symptoms Among Men.” International Journal of Eating Disorders 52, no. 8: 871–884. 10.1002/eat.23123. 31228298

[eat70080-bib-0014] George, D. , and P. Mallery . 2016. IBM SPSS Statistics 23 Step by Step: A Simple Guide and Reference. Fourteenth ed. Routledge, Taylor & Francis Group.

[eat70080-bib-0015] Griffiths, S. , J. M. Mond , Z. Li , et al. 2015. “Self‐Stigma of Seeking Treatment and Being Male Predict an Increased Likelihood of Having an Undiagnosed Eating Disorder: Predicting Undiagnosed Eating Disorders.” International Journal of Eating Disorders 48, no. 6: 775–778. 10.1002/eat.22413. 26052695

[eat70080-bib-0016] Gurney, V. W. , and K. A. Halmi . 2001. “An Eating Disorder Curriculum for Primary Care Providers.” International Journal of Eating Disorders 30, no. 2: 209–212. 10.1002/eat.1074. 11449455

[eat70080-bib-0017] Halbeisen, G. , N. Laskowski , G. Brandt , U. Waschescio , and G. Paslakis . 2024. “Eating Disorders in Men—An Underestimated Problem, an Unseen Need.” Deutsches Ärzteblatt International 121: 86–91. 10.3238/arztebl.m2023.0246. 38019152 PMC11002438

[eat70080-bib-0018] Hellen, N. W. T. 2025. “MISSR: Classify Missing Data as MCAR, MAR, or MNAR.” https://github.com/NoahHellen/missr.

[eat70080-bib-0020] Krauth, C. , K. Buser , and H. Vogel . 2002. “How High Are the Costs of Eating Disorders—Anorexia Nervosa and Bulimia Nervosa—For German Society?” European Journal of Health Economics 3, no. 4: 244–250. 10.1007/s10198-002-0137-2. 15609150

[eat70080-bib-0021] Kuznetsova, A. , P. B. Brockhoff , and R. H. B. Christensen . 2017. “lmerTest Package: Tests in Linear Mixed Effects Models.” Journal of Statistical Software 82, no. 13: 1–26. 10.18637/jss.v082.i13.

[eat70080-bib-0022] Lehe, M. S. , G. Halbeisen , V. C. Juergensen , L. Sabel , S. Steins‐Loeber , and G. Paslakis . 2025. “Boys Don't Try? Gendered Stigma Specifically Reduces Help‐Seeking for Disordered Eating in Men, but Not Women.” Journal of Eating Disorders 13, no. 1: 204. 10.1186/s40337-025-01407-7. 40963138 PMC12442273

[eat70080-bib-0023] Lehe, M. S. , G. Halbeisen , and G. Paslakis . 2025. “Intervention Against the Stigmatization of Men With Eating Disorders in Primary Care (iSMEsH): Protocol for a Randomized Mixed‐Methods Evaluation Trial.” PLoS One 20, no. 10: e0333997. 10.1371/journal.pone.0333997. 41066335 PMC12510528

[eat70080-bib-0024] Lehe, M. S. , G. Halbeisen , S. Steins‐Loeber , and G. Paslakis . 2024. “Invisible Walls? Stigma‐Related Perceptions Are Associated With Reduced Help‐Seeking Intentions for Disordered Eating in Men.” Journal of Eating Disorders 12, no. 1: 200. 10.1186/s40337-024-01152-3. 39633398 PMC11619332

[eat70080-bib-0025] Lehe, M. S. , L. Sabel , G. Halbeisen , and G. Paslakis . 2025. Intervention Against The Stigmatization of Men With Eating Disorders in Primary Care: Video‐Based Training Modules [Dataset]. 10.60517/n583xw963. PMC12510528 41066335

[eat70080-bib-0026] Link, B. G. , and J. C. Phelan . 2001. “Conceptualizing Stigma.” Annual Review of Sociology 27: 363–385. 10.1146/annurev.soc.27.1.363.

[eat70080-bib-0027] Mangweth‐Matzek, B. 2022. “Herausforderung Gender und Essstörungen: Essstörung ist nicht (nur) weiblich.” PiD—Psychotherapie Im Dialog 23, no. 1: 34–37. 10.1055/a-1477-1110.

[eat70080-bib-0028] Modgill, G. , S. B. Patten , S. Knaak , A. Kassam , and A. C. Szeto . 2014. “Opening Minds Stigma Scale for Health Care Providers (OMS‐HC): Examination of Psychometric Properties and Responsiveness.” BMC Psychiatry 14, no. 1: 120. 10.1186/1471-244X-14-120. 24758158 PMC4024210

[eat70080-bib-0029] Mycock, G. , U. Foye , C. Edwards , and G. Molnár . 2025. “Men's Formal Help‐Seeking for Eating and/or Body Image Psychopathology: A Systematic Review of Barriers and Facilitators.” Journal of Men's Studies 34, no. 1: 45–77. 10.1177/10608265251336747.

[eat70080-bib-0049] Neubauer, K. , A. Weigel , A. Daubmann , et al. 2014. “Paths to First Treatment and Duration of Untreated Illness in Anorexia Nervosa: Are There Differences According to Age of Onset?” European Eating Disorders Review 22, no. 4: 292–298. 10.1002/erv.2300. 24888519

[eat70080-bib-0030] R Core Team . 2025. R: A Language and Environment for Statistical Computing. R Foundation for Statistical Computing. https://www.R‐project.org/.

[eat70080-bib-0031] Räisänen, U. , and K. Hunt . 2014. “The Role of Gendered Constructions of Eating Disorders in Delayed Help‐Seeking in Men: A Qualitative Interview Study.” BMJ Open 4, no. 4: 004342. 10.1136/bmjopen-2013-004342. PMC3987710 24713213

[eat70080-bib-0032] Richardson, C. , and G. Paslakis . 2021. “Men's Experiences of Eating Disorder Treatment: A Qualitative Systematic Review of Men‐Only Studies.” Journal of Psychiatric and Mental Health Nursing 28, no. 2: 237–250. 10.1111/jpm.12670. 32608115

[eat70080-bib-0033] Ruffo, E. S. , K. K. Soylemez , and J. Lusher . 2025. “Masculinity, Body Image and Eating Pathology: A Review of Eating Disorders Amongst Men.” GSC Advanced Research and Reviews 23, no. 1: 34–38. 10.30574/gscarr.2025.23.1.0104.

[eat70080-bib-0034] Rüsch, N. , M. C. Angermeyer , and P. W. Corrigan . 2005. “Das Stigma Psychischer Erkrankung: Konzepte, Formen und Folgen.” Psychiatrische Praxis 32, no. 5: 221–232. 10.1055/s-2004-834566. 15983885

[eat70080-bib-0035] Rüsch, N. , M. Heland‐Graef , and J. Berg‐Peer . 2020. Das Stigma Psychischer Erkrankung: Strategien gegen Ausgrenzung und Diskriminierung: wissenschaftsbasiertes Sachbuch. 1st ed. Elsevier.

[eat70080-bib-0036] Schielzeth, H. , N. J. Dingemanse , S. Nakagawa , et al. 2020. “Robustness of Linear Mixed‐Effects Models to Violations of Distributional Assumptions.” Methods in Ecology and Evolution 11, no. 9: 1141–1152. 10.1111/2041-210X.13434.

[eat70080-bib-0037] Schwarzer, R. , and M. Jerusalem . 1995. “Generalized Self‐Efficacy Scale.” In Measures in Health Psychology: A User's Portfolio. Causal and Control Beliefs, edited by J. Weinman , S. Wright , and M. Johnston , 35–37. NFER‐NELSON.

[eat70080-bib-0038] Schwarzer, R. , and M. Jerusalem . 1999. Skalen zur Erfassung von Lehrer‐ und Schülermerkmalen. Dokumentation der psychometrischen Verfahren im Rahmen der Wissenschaftlichen Begleitung des Modellversuchs Selbstwirksame Schulen. Freie Universität Berlin.

[eat70080-bib-0039] Sofyan, Z. , and C. Meinel . 2024a. “Exploring Interactive Content in MOOCs for Training Educators in AI Education.” In 2024International Conference on Electrical Engineering and Informatics (ICELTICs), 51–55. 10.1109/ICELTICs62730.2024.10776485.

[eat70080-bib-0040] Sofyan, Z. , and C. Meinel . 2024b. “Should We Use It or Not? Insights Into Utilizing Interactive Content in AI‐Teaching MOOCs Platform.” In 2024IEEE Digital Education and MOOCS Conference (DEMOcon), 1–6. 10.1109/DEMOcon63027.2024.10748215.

[eat70080-bib-0041] Sommet, N. , D. L. Weissman , N. Cheutin , and A. J. Elliot . 2023. “How Many Participants Do I Need to Test an Interaction? Conducting an Appropriate Power Analysis and Achieving Sufficient Power to Detect an Interaction.” Advances in Methods and Practices in Psychological Science 6, no. 3: 25152459231178728. 10.1177/25152459231178728.

[eat70080-bib-0042] Statistisches Bundesamt . 2021. Tiefgegliederte Diagnosedaten der Krankenhauspatientinnen und ‐patienten (Datensatzstruktur), 2019. Statistisches Bundesamt.

[eat70080-bib-0043] Steinert, C. , K. Stadter , R. Stark , and F. Leichsenring . 2017. “The Effects of Waiting for Treatment: A Meta‐Analysis of Waitlist Control Groups in Randomized Controlled Trials for Social Anxiety Disorder.” Clinical Psychology & Psychotherapy 24, no. 3: 649–660. 10.1002/cpp.2032. 27445199

[eat70080-bib-0044] Stuart, H. , S.‐P. Chen , R. Christie , et al. 2014. “Opening Minds in Canada: Background and Rationale.” Canadian Journal of Psychiatry. Revue Canadienne de Psychiatrie 59, no. 10 Suppl 1: S8–S12. 10.1177/070674371405901s04. 25565705 PMC4213755

[eat70080-bib-0045] Treasure, J. , T. A. Duarte , and U. Schmidt . 2020. “Eating Disorders.” Lancet 395, no. 10227: 899–911. 10.1016/S0140-6736(20)30059-3. 32171414

[eat70080-bib-0046] Udo, T. , and C. M. Grilo . 2019. “Psychiatric and Medical Correlates of DSM‐5 Eating Disorders in a Nationally Representative Sample of Adults in the United States.” International Journal of Eating Disorders 52, no. 1: 42–50. 10.1002/eat.23004. 30756422

[eat70080-bib-0019] UK Parliament . 2019. “Ignoring the Alarms Follow‐Up: Too Many Avoidable Deaths From Eating Disorders.” https://publications.parliament.uk/pa/cm201719/cmselect/cmpubadm/855/85502.htm.

[eat70080-bib-0047] Wickham, H. 2016. GGPLOT2: Elegant Graphics for Data Analysis. Springer International Publishing. 10.1007/978-3-319-24277-4.

[eat70080-bib-0048] Zuaboni, G. , T. Elmer , F. Rabenschlag , et al. 2021. “Psychometric Evaluation of the German Version of the Opening Minds Stigma Scale for Health Care Providers (OMS‐HC).” BMC Psychology 9, no. 1: 86. 10.1186/s40359-021-00592-9. 34016166 PMC8139058

