# Supplementary material for: Efficacy of the Intervention Against the Stigmatization of Men With Eating Disorders in Primary Healthcare (iSMEsH): Results From a Randomized Waitlist‐Controlled Study
Source: Int J Eat Disord. 2026 Mar 9;59(7):1517–30. doi: 10.1002/eat.70080 (PMC13326786; doi:10.1002/eat.70080)
Supplement: Supplementary file 1 — Data S1: eat70080‐sup‐0001‐Supinfo.docx. [file EAT-59-1517-s001.docx]

# Supporting Information 1

## Supplementary Text 1: Recruitment and Participant Engagement

Recruitment occurred nationwide between July 23, 2024 and May 10, 2025 using a multi-channel approach (printed leaflets, personal referrals, and mass-email invitations) and a low-burden online self-enrolment pathway. Interested participants could self-register via a webform on the project website, which was consistently provided as a link and QR code in all recruitment materials.

To reach general practitioners (GPs), we sourced contact information via extensive manual online searches, webscraping of local/regional physician networks, and contact registers of medical professional organizations. This approach was complemented by a probabilistic nationwide sample of 10,000 GPs drawn from the federal register of physicians (Bundesarztregister, Kassenärztliche Bundesvereinigung). Medical students in their final clinical training year were recruited through local university networks and mass emailing via clinical training year coordinators at medical faculties across Germany.

Participant engagement was supported through procedures designed to minimize burden and fit clinical schedules: the training was delivered remotely via Moodle and could be completed self-paced within a 14-day access period. To support completion, participants received reminder emails at predefined time points during the participation window (i.e., reminders after 7 days for pending surveys, and at the midpoint and 2 days before the end of the training access period). Engagement and completion were further incentivized through a certificate of participation, access to downloadable supporting materials, and—among GPs—reimbursement and a Continuing Medical Education (CME) credit point.

Patient and public involvement supported dissemination. Men with lived experience of eating disorders and GPs from the project’s public engagement advisory board advised on suitable access channels and communication strategies and disseminated recruitment materials within relevant networks to facilitate outreach to the target group.

Feasibility and implementation outcomes of the project will be reported in a separate publication.

**References**

Lehe, M. S., Halbeisen, G., & Paslakis, G. (2025). Intervention against the stigmatization of men with eating disorders in primary care (iSMEsH): Protocol for a randomized mixed-methods evaluation trial. *PLOS ONE*, *20*(10), e0333997. https://doi.org/10.1371/journal.pone.0333997

## Supplementary Text 2: Measurement Instruments

Primary outcomes covered cognitive, affective, and behavioral facets of men’s eating disorder-related stigma. For more details, please refer to the published study protocol (Lehe et al., 2025).

**Cognitive facet**

The cognitive facet of stigma (i.e., stereotypes, misinformation, knowledge deficits) was operationalized as knowledge about eating disorders (EDs) in boys and men, and assessed using ten self-developed single-choice items (one correct response per item; see example item in the box below). The items were developed specifically for the iSMEsH intervention. Item content was informed by a thorough literature review and refined through iterative discussion within our multidisciplinary team of experienced clinicians and the project advisory board of men with lived experience of an ED. The final item set was designed to capture clinically relevant knowledge gaps related to EDs in boys and men (e.g., typical symptom presentation, risk factors, and gender-related stereotypes) and was aligned with the intervention’s educational content. The items were piloted in an independent sample of medical students prior to the study to evaluate comprehensibility and item performance. As part of the Continuing Medical Education (CME) accreditation procedure, the intervention and accompanying knowledge assessment underwent a formal review by the regional medical chamber Westfalen-Lippe (Landesärztekammer Westfalen-Lippe; ÄKWL). A total knowledge score was computed as the sum of correct responses (range: 0–10), with higher scores indicating greater knowledge about EDs in boys and men.

**Example item**

*Which of the following statements about men and eating disorders is correct?*

a. The symptoms of eating disorders present almost identically in women and men.

b. The prevalence of eating disorders in men has been increasing more strongly than in women in recent years. [correct response]

c. Eating disorders affect only gay men.

d. Eating disorders occur in men and women at approximately the same rate.

e. Eating disorders also occur in men, but are usually only of subclinical relevance in men.

**Affective facet**

The affective facet of stigma (i.e., prejudice, negative attitudes) was assessed using an adaption of the validated German version of the Opening Minds Stigma Scale for Health Care Providers (OMS-HC; Modgill et al., 2014; Zuaboni et al., 2021). The OMS-HC consists of 15 self-report items rated on a 5-point Likert scale from 1 (*strongly disagree*) to 5 (*strongly agree*) and is designed to capture stigmatizing attitudes among healthcare professionals. For the present trial, the wording of the items was adapted to specifically refer to men with EDs by replacing the original target group phrasing (“people/person with a mental illness”) with “men with an eating disorder”. The scale comprises three subscales (‘attitudes’, ‘disclosure and help-seeking’, and ‘social distance’). We used the global scale mean score as the primary index of affective stigma, with higher scores indicating more stigmatizing attitudes. Internal consistency was acceptable across measurement time points (GPs: .70 ≤ α ≤ .79; medical students: .63 ≤ α ≤ .82). Our adapted scale demonstrated acceptable model fit in confirmatory factor analyses conducted separately for general practitioners and medical students.

**Behavioral facet**

The behavioral facet of stigma (i.e., discrimination/unequal treatment) was operationalized as perceived self-efficacy in identifying and managing eating disorder symptoms in boys and men. Treatment self-efficacy was assessed using three carefully selected items from the German version of the General Self-Efficacy Scale (GSE) (Schwarzer & Jerusalem, 1995, 1999). The selection followed similar scientific studies in the field of anti-stigma training for medical professionals (e.g., <https://base-elearning.de>) and was performed upon extensive discussion of the face validity of selected items. Additionally, the items were adapted to the study context of treating men with eating disorders (“I can remain calm when facing difficulties in the treatment of men with eating disorders because I can rely on my coping abilities”, “I am confident that I could deal efficiently with unexpected events in the treatment of men with eating disorders”, “When I am confronted with a man with an eating disorder, I can usually find several solutions”). The adaptation process was informed by extensive discussion within the research team, including experienced clinicians, and by feedback from the advisory board comprising men with lived experience of an ED and general practitioners. Responses were recorded on a 4-point scale ranging from 1 (*not at all true*) to 4 (*exactly true*). A total score was computed by summing the three items, with higher scores indicating greater self-efficacy. The adapted three-item scale showed acceptable internal consistency across measurement occasions (GPs: .76 ≤ α ≤ .86; medical students: .72 ≤ α ≤ .85).

**References**

Lehe, M. S., Halbeisen, G., & Paslakis, G. (2025). Intervention against the stigmatization of men with eating disorders in primary care (iSMEsH): Protocol for a randomized mixed-methods evaluation trial. *PLOS ONE*, *20*(10), e0333997. https://doi.org/10.1371/journal.pone.0333997

Modgill, G., Patten, S. B., Knaak, S., Kassam, A., & Szeto, A. C. (2014). Opening Minds Stigma Scale for Health Care Providers (OMS-HC): Examination of psychometric properties and responsiveness. *BMC Psychiatry*, *14*(1), 120. https://doi.org/10.1186/1471-244X-14-120

Schwarzer, R., & Jerusalem, M. (1995). Generalized Self-Efficacy scale. In J. Weinman, S. Wright, & M. Johnston (Eds.), *Measures in health psychology: A user’s portfolio. Causal and control beliefs* (pp. 35–37). NFER-NELSON.

Schwarzer, R., & Jerusalem, M. (Eds.). (1999). *Skalen zur Erfassung von Lehrer- und Schülermerkmalen. Dokumentation der psychometrischen Verfahren im Rahmen der Wissenschaftlichen Begleitung des Modellversuchs Selbstwirksame Schulen*. Freie Universität Berlin.

Zuaboni, G., Elmer, T., Rabenschlag, F., Heumann, K., Jaeger, S., Kozel, B., Mahlke, C. I., Theodoridou, A., Jaeger, M., & Rüsch, N. (2021). Psychometric evaluation of the German version of the Opening Minds Stigma Scale for Health Care Providers (OMS-HC). *BMC Psychology*, *9*(1), 86. https://doi.org/10.1186/s40359-021-00592-9

## Supplementary Text 3: Per-protocol Analyses

**Statistical analysis**

The per-protocol analyses for each primary outcome were conducted separately for GP and medical student samples as 2 (group: IG vs. waitlist CG) × 3 (time: pre vs. post vs. follow-up) mixed ANOVAs, with case-wise exclusion for missing data (i.e., we included only complete datasets). The ANOVAs were conducted with *aov_ez()* from R package *afex* 1.4.1 (Singmann et al., 2024), using *emmeans* 1.11.2 (Lenth, 2025) for Bonferroni-adjusted pairwise comparisons. All other reportings adhere to the specifications stated in the methods section of the main manuscript.

**General practitioners**

The 2 (group) × 3 (time) mixed ANOVAs revealed the expected two-way interactions across outcomes (see **Table S1** for effects, **Table S2** for descriptive statistics), all *F*s ≥ 3.42, all *p*s ≤ .035, all η_p_² ≥ .04.

As expected, GPs in the IG gained knowledge from baseline to post-intervention, *p*_T1-T2_ < .001, and knowledge remained stable at follow up, *p*_T2-T3_ > .999. GPs in the waitlist condition showed no differences between baseline and pre-intervention assessment, *p*_T1-T2_ > .999, but gained in knowledge over the intervention, *p*_T2-T3_ = .002. Viewed differently, GPs in intervention and control conditions did not differ at baseline or at study end, *ps* ≥ .590, and intervention GPs showed the expected advantage over waitlist GPs immediately after they had completed (while the waitlist GPs had not yet completed) the training, *p* < .001.

Affective stigma displayed a similar trend, though not all anticipated effects were significant. The IG decline in stigmatizing attitudes did not reach significance over the intervention period, *p*_T1-T2_ > .999, or at follow-up, *p*_T2-T3_ = .505. In contrast, the waitlist CG showed a significant decrease in stigmatizing attitudes over the waiting period, *p*_T1-T2_ = .012, that persisted to follow-up, *p*_T1-T3_ < .002, with no significant further decline over the intervention participation, *p*_T2-T3_ > .999. Both GP groups did not differ significantly from each other across timepoints, *ps* ≥ .058.

Finally, regarding treatment self-efficacy, data yielded the full expected pattern. GPs in the IG gained self-efficacy over the intervention period, *p*_T1-T2_ = .001, which persisted to follow-up, *p*_T2-T3_ = .797. Waitlist CG participants did not report self-efficacy gains over the waiting period, *p*_T1-T2_ = .278, but profited from the intervention period, *p*_T2-T3_ < .001. Respectively, groups differed at T2 after the IG received treatment first, *p* < .001, but not at baseline or study end, *p* ≥ .156.

**Medical students**

In the medical students’ sample, the 2 (group) × 3 (time) mixed ANOVAs confirmed the anticipated two-way interactions across outcomes (see **Table S1** for effects, **Table S2** for descriptive statistics), all *F*s ≥ 4.77, all *p*s ≤ .009, all η_p_²s ≥ .04.

As anticipated, students in the IG demonstrated a significant increase in knowledge from baseline to post-intervention, *p*_T1-T2_ < .001, which remained stable at follow-up, *p*_T2-T3_ > .999. Students in the waitlist CG showed no increase in knowledge from baseline to pre-intervention, *p*_T1-T2_ = .420, and furthermore in line with expectations, exhibited a further knowledge gain following the intervention, *p*_T2-T3_ < .001. In line with our hypotheses, there were no significant differences between the IG and the waitlist CG either at baseline or at the end of the study, *p*s ≥ .271, whilst the IG had a temporary advantage over the waitlist CG immediately after completing the training, *p* < .001.

Regarding affective stigma, the student IG neither exhibited the expected decline over the course of the intervention, nor at follow-up, *ps* ≥ .702. However, the waitlist CG, as expected, demonstrated no decline over the waiting period, *p*_T1-T2_ = .254, but after intervention participation, *p*_T2-T3_ < .001, however not compared to baseline, *p*_T1-T3_ = .267. As such, groups did differ in stigmatizing attitudes at study end, *p* = .026, but not at baseline and T2, *ps* ≥ .231.

With respect to treatment self-efficacy, the students' data revealed the anticipated pattern in full. IG participants gained self-efficacy over their participation, *p*_T1-T2_ < .001, with gains persisting unaltered at follow-up, *p*_T2-T3_ > .999. Waitlist participants did not report gains in self-efficacy over the waiting period, *p*_T1-T2_ > .999, but profited from the intervention, *p*_T2-T3_ < .001. Subsequently, groups did not differ at baseline and study end, *ps* ≥ .207, but the IG participants reported higher self-efficacy compared to CG participants right after their participation, *p* < .001.

**References**

Lenth, R. V. (2025). *emmeans: Estimated Marginal Means, aka Least-Squares Means*. https://doi.org/10.32614/CRAN.package.emmeans

Singmann, H., Bolker, B., Westfall, J., Aust, F., & Ben-Shachar, M. S. (2024). *afex: Analysis of Factorial Experiments*. https://doi.org/10.32614/CRAN.package.afex

**Table S1**

*ANOVA Fixed Effects for Stigma Facet Outcomes by Population (Per-Protocol Analyses)*

| *Sample* | *DV* | *Group* | *Time* | *Group × Time* |
| --- | --- | --- | --- | --- |
| GPs | Cognition | *F*(1, 95) = 7.97, *p* = .006**, η_p_² = .08 | *F*(2, 190) = 10.22, *p* < .001***, η_p_² = .10 | *F*(2, 190) = 5.46, *p* = .005**, η_p_² = .05 |
|  | Affect | *F*(1, 95) = 0.00, *p* = .958, η_p_² = .00 | *F*(2, 190) = 4.34, *p* = .014*, η_p_² = .04 | *F*(2, 190) = 3.42, *p* = .035*, η_p_² = .04 |
|  | Behavior | *F*(1, 95) = 10.83, *p* = .001***, η_p_² = .10 | *F*(2, 190) = 20.99, *p* < .001***, η_p_² = .18 | *F*(2, 190) = 19.14, *p* < .001***, η_p_² = .17 |
| Students | Cognition | *F*(1, 110) = 4.70, *p* = .032*, η_p_² = .04 | *F*(2, 220) = 22.86, *p* < .001***, η_p_² = .17 | *F*(2, 220) = 15.23, *p* < .001***, η_p_² = .12 |
|  | Affect | *F*(1, 109) = 0.86, *p* = .357, η_p_² = .01 | *F*(2, 218) = 0.99, *p* = .373, η_p_² = .01 | *F*(2, 218) = 4.77, *p* = .009**, η_p_² = .04 |
|  | Behavior | *F*(1, 110) = 4.32, *p* = .040*, η_p_² = .04 | *F*(2, 220) = 41.95, *p* < .001***, η_p_² = .28 | *F*(2, 220) = 11.85, *p* < .001***, η_p_² = .10 |

*Note*. Results are from mixed ANOVAs with case-wise exclusion for missing data. DV = Dependent Variable. GPs = General Practitioners. Students = Medical Students. Cognition = Continuing Medical Education (CME) Knowledge Items. Affect = Opening Minds Stigma Scale for Healthcare Providers (OMS-HC). Behavior = General Self-Efficacy Scale (GSE).
**p* < .05. ***p* < .01. ****p* < .001.

**Table S2**

*Descriptive Statistics by Population, Timepoint, and Group for Stigma Facet Outcomes (Per-Protocol Analyses)*

|  |  |  | *Total* | | *Intervention Group* | | *Control Group* | |
| --- | --- | --- | --- | --- | --- | --- | --- | --- |
| *Sample* | *DV* | *Timepoint* | *N of completers* | *M ± SD* | *N of completers* | *M ± SD* | *N of completers* | *M ± SD* |
| GPs | Cognition | T1 | 97 | 8.39 ± 1.34 | 45 | 8.42 ± 1.22_a_ | 52 | 8.37 ± 1.46_a_ |
|  |  | T2 | 97 | 8.85 ± 1.24 | 45 | 9.40 ± 0.81_a_ | 52 | 8.37 ± 1.34_b_ |
|  |  | T3 | 97 | 9.14 ± 1.01 | 45 | 9.21 ± 1.06_a_ | 52 | 9.08 ± 0.97_a_ |
|  | Affect | T1 | 97 | 1.95 ± 0.37 | 45 | 1.87 ± 0.38_a_ | 52 | 2.01 ± 0.36_a_ |
|  |  | T2 | 97 | 1.79 ± 0.43 | 45 | 1.80 ± 0.47_a_ | 52 | 1.78 ± 0.40_a_ |
|  |  | T3 | 97 | 1.85 ± 0.42 | 45 | 1.92 ± 0.47_a_ | 52 | 1.77 ± 0.36_a_ |
|  | Behavior | T1 | 97 | 7.70 ± 1.97 | 45 | 7.82 ± 1.81_a_ | 52 | 7.60 ± 2.12_a_ |
|  |  | T2 | 97 | 8.08 ± 2.24 | 45 | 9.38 ± 1.43_a_ | 52 | 6.96 ± 2.22_b_ |
|  |  | T3 | 97 | 9.22 ± 1.31 | 45 | 9.02 ± 1.39_a_ | 52 | 9.40 ± 1.21_a_ |
| Students | Cognition | T1 | 112 | 8.61 ± 1.03 | 42 | 8.50 ± 1.02_a_ | 70 | 8.67 ± 1.03_a_ |
|  |  | T2 | 112 | 8.80 ± 1.25 | 42 | 9.48 ± 0.71_a_ | 70 | 8.40 ± 1.33_b_ |
|  |  | T3 | 112 | 9.47 ± 0.68 | 42 | 9.38 ± 0.73_a_ | 70 | 9.53 ± 0.65_a_ |
|  | Affect | T1 | 111 | 1.73 ± 0.29 | 42 | 1.75 ± 0.28_a_ | 69 | 1.72 ± 0.30_a_ |
|  |  | T2 | 111 | 1.79 ± 0.37 | 42 | 1.74 ± 0.42_a_ | 69 | 1.83 ± 0.34_a_ |
|  |  | T3 | 111 | 1.70 ± 0.45 | 42 | 1.82 ± 0.62_a_ | 69 | 1.62 ± 0.29_b_ |
|  | Behavior | T1 | 112 | 7.02 ± 1.81 | 42 | 6.74 ± 2.00_a_ | 70 | 7.19 ± 1.68_a_ |
|  |  | T2 | 112 | 7.95 ± 2.21 | 42 | 9.02 ± 1.32_a_ | 70 | 7.30 ± 2.39_b_ |
|  |  | T3 | 112 | 9.10 ± 1.32 | 42 | 9.12 ± 1.60_a_ | 70 | 9.09 ± 1.14_a_ |

*Note*. Means with different subscript letters are statistically different at *p* < .05 of a Bonfferoni-adjusted pairwise comparison Student’s t-test. DV = Dependent Variable. GPs = General Practitioners. Students = Medical Students. Cognition = Continuing Medical Education (CME) Knowledge Items. Affect = Opening Minds Stigma Scale for Healthcare Providers (OMS-HC). Behavior = General Self-Efficacy Scale (GSE).

**Table S3**

*Adherence by Group for General Practitioner and Medical Student Samples*

|  | Total | | General Practitioners | | Medical Students | |
| --- | --- | --- | --- | --- | --- | --- |
|  | IG | Waitlist CG | IG | Waitlist CG | IG | Waitlist CG |
| Variable | *M* (*SD*), *n* (%) | *M* (*SD*), *n* (%) | *M* (*SD*), *n* (%) | *M* (*SD*), *n* (%) | *M* (*SD*), *n* (%) | *M* (*SD*), *n* (%) |
| Number of completed modules | 3.77 (2.54) | 3.11 (2.70) | 4.04 (2.57) | 3.05 (2.74) | 3.44 (2.43) | 3.16 (2.68) |
| Participants |  |  |  |  |  |  |
| randomized (% of population) | 168 (40.38%) | 248 (59.62%) | 80 (44.44%) | 100 (55.56%) | 88 (37.29%) | 148 (62.71%) |
| dropped out (% of all randomized to group) | 55 (32.74%) | 112 (45.16%) | 23 (28.75%) | 44 (44.00%) | 32 (36.36%) | 68 (45.95%) |
| completed modules 1-4, irrespective of post assessment (% of all randomized to group) | 113 (67.26%) | 136 (54.84%) | 57 (71.25%) | 56 (56.00%) | 56 (63.63%) | 80 (54.05%) |
| completed modules 1-4, and pre & post assessments (% of all randomized to group) | 87 (51.79%) | 122 (49.19%) | 45 (56.25%) | 52 (52.00%) | 42 (47.73%) | 70 (47.30%) |
| Completers per module (*n,* % of all randomized to group) |  |  |  |  |  |  |
| Module 1 | 123 (73,21%) | 151 (60,89%) | 60 (75,00%) | 59 (59,00%) | 63 (71,59%) | 92 (62,16%) |
| Module 2 | 118 (70,24%) | 144 (58,06%) | 57 (71,25%) | 56 (56,00%) | 61 (69,32%) | 88 (59,56%) |
| Module 3 | 115 (68,45%) | 141 (56,85%) | 57 (71,25%) | 56 (56,00%) | 58 (65,91%) | 85 (57,43%) |
| Module 4 | 113 (67,26%) | 136 (54,84%) | 57 (71,25%) | 56 (56,00%) | 56 (63,64%) | 80 (54,05%) |
| Module 5 | 84 (50.000%) | 110 (44,35%) | 48 (60,00%) | 43 (43,00%) | 36 (40,91%) | 67 (45,27%) |
| Module 6 | 71 (42,26%) | 87 (35,08%) | 44 (55,00%) | 35 (35,00%) | 27 (30,68%) | 52 (35,14%) |

*Note*. IG = intervention group. CG = control group.

**Table S4**

*Extended Participant Characteristics for General Practitioner and Medical Student Samples as Randomized*

|  | General Practitioners | | |  | Medical Students | | |
| --- | --- | --- | --- | --- | --- | --- | --- |
|  | Total  (*n* = 130) | Intervention group  (*n* = 68) | Control group  (*n* = 62) |  | Total  (*n* = 162) | Intervention group  (*n* = 68) | Control group  (*n* = 94) |
| *Variable* | *M* ± *SD* / *n* (%) | *M* ± *SD* / *n* (%) | *M* ± *SD* / *n* (%) |  | *M* ± *SD* / *n* (%) | *M* ± *SD* / *n* (%) | *M* ± *SD* / *n* (%) |
| Employment situation^a^ |  |  |  |  |  |  |  |
| Employed | 122 (94.57) | 64 (95.52) | 58 (93.55) |  | - | - | - |
| Retired | 0 (0.0) | 0 (0.0) | 0 (0.0) |  | - | - | - |
| Unemployed | 2 (1.55) | 0 (0.0) | 2 (3.23) |  | - | - | - |
| Homemaker/Caregiver | 2 (1.55) | 1 (1.49) | 1 (1.61) |  | - | - | - |
| Other | 3 (2.33) | 2 (2.99) | 1 (1.61) |  | - | - | - |
| Employment duration (in years) (in medical students: Duration of training year placement (in months))^b^ | 14.42 ± 10.88 | 15.34 ± 11.50 | 13.42 ± 10.17 |  | 5.51 ± 3.58 | 5.70 ± 3.50 | 5.37 ± 3.65 |
| Specialty area (in medical students: specialty area of current training year quadrimester)^c^ |  |  |  |  |  |  |  |
| General Medicine | 115 (68.45) | 60 (68.18) | 55 (68.75) |  | 4 (2.48) | 1 (1.49) | 3 (3.19) |
| Pediatrics | 2 (1.19) | 1 (1.14) | 1 (1.25) |  | 10 (6.21) | 8 (11.94) | 2 (2.13) |
| Gynecology | 1 (0.60) | 0 (0.00) | 1 (1.25) |  | 3 (1.86) | 2 (2.99) | 1 (1.06) |
| Internal Medicine | 30 (17.86) | 15 (17.05) | 15 (18.75) |  | 57 (35.40) | 21 (31.34) | 36 (38.30) |
| Psychiatry/ Psychosomatic Medicine/ Psychotherapy | 7 (4.17) | 3 (3.41) | 4 (5.00) |  | 13 (8.07) | 6 (8.96) | 7 (7.45) |
| Emergency Medicine | 6 (3.57) | 5 (5.68) | 1 (1.25) |  | 1 (0.62) | 0 (0.00) | 1 (1.06) |
| Surgery | 0 (0.0) | 0 (0.0) | 0 (0.0) |  | 48 (29.81) | 19 (28.36) | 29 (30.85) |
| Neurology | 0 (0.0) | 0 (0.0) | 0 (0.0) |  | 6 (3.73) | 1 (1.49) | 5 (5.32) |
| Other | 7 (4.17) | 4 (4.55) | 3 (3.75) |  | 19 (11.80) | 9 (13.43) | 10 (10.64) |
| Duration within specialty area (years)^d^ |  |  |  |  |  |  |  |
| General Medicine | 8.90 ± 9.75 | 10.48 ± 10.69 | 7.16 ± 8.36 |  | - | - | - |
| Pediatrics | 6.50 ± 7.78 | 1.00 ± 0.00 | 12.00 ± 0.00 |  | - | - | - |
| Gynecology | 2.00 ± 0.00 | 0 ± 0.00 | 2.00 ± 0.00 |  | - | - | - |
| Internal Medicine | 14.40 ± 11.15 | 16.33 ± 11.92 | 12.47 ± 10.36 |  | - | - | - |
| Psychiatry/ Psychosomatic Medicine/ Psychotherapy | 11.25 ± 12.44 | 4.75 ± 6.40 | 17.75 ± 15.50 |  | - | - | - |
| Emergency Medicine | 10.67 ± 3.93 | 10.40 ± 4.34 | 12.00 ± 0.00 |  | - | - | - |
| Surgery | 0 ± 0.00 | 0 ± 0.00 | 0 ± 0.00 |  | - | - | - |
| Neurology | 0 ± 0.00 | 0 ± 0.00 | 0 ± 0.00 |  | - | - | - |
| Other | 9.57 ± 9.36 | 8.00 ± 9.76 | 11.67 ± 10.41 |  | - | - | - |
| Duration in current training year quadrimester (months)^e^ | - | - | - |  | 1.94 ± 1.13 | 1.78 ± 1.01 | 2.06 ± 1.20 |
| Duration of study (semesters)^f^ | - | - | - |  | 12.14 ± 1.52 | 11.96 ± 1.35 | 12.27 ± 1.62 |
| Currently practicing as general practitioner (in medical students: Currently in training in general practice)^g^ |  |  |  |  |  |  |  |
| Yes | 110 (85.27) | 58 (86.57) | 52 (83.87) |  | 5 (3.11) | 2 (2.99) | 3 (3.19) |
| No | 19 (14.73) | 9 (13.43) | 10 (16.13) |  | 156 (96.89) | 65 (97.01) | 91 (96.81) |
| Experience in a psychiatric/ psychological job (in medical students: Quadrimester completed in psychiatric/ psychological setting)^h^ |  |  |  |  |  |  |  |
| Yes | 49 (37.98) | 29 (43.28) | 20 (32.26) |  | 24 (14.91) | 9 (13.43) | 15 (15.96) |
| No | 80 (62.02) | 38 (56.72) | 42 (67.74) |  | 110 (69.32) | 49 (73.13) | 61 (64.89) |
| No, but planned | - | - | - |  | 27 (16.77) | 9 (13.43) | 18 (19.15) |
| Desired future specialty area^i^ |  |  |  |  |  |  |  |
| General Medicine | - | - | - |  | 28 (17.39) | 12 (17.91) | 16 (17.02) |
| Don‘ know yet | - | - | - |  | 24 (14.91) | 10 (14.93) | 14 (14.89) |
| Pediatrics | - | - | - |  | 13 (8.07) | 9 (13.43) | 4 (4.26) |
| Gynecology | - | - | - |  | 7 (4.35) | 3 (4.48) | 4 (4.26) |
| Internal Medicine | - | - | - |  | 17 (10.56) | 7 (10.45) | 10 (10.64) |
| Psychiatry / Psychosomatic medicine / Psychotherapy | - | - | - |  | 35 (21.74) | 13 (19.40) | 22 (23.40) |
| Emergency Medicine | - | - | - |  | 2 (1.24) | 0 (0.00) | 2 (2.13) |
| Surgery | - | - | - |  | 7 (4.35) | 5 (7.46) | 2 (2.13) |
| Neurology | - | - | - |  | 8 (4.97) | 3 (4.48) | 5 (5.32) |
| Other | - | - | - |  | 20 (12.42) | 5 (7.46) | 15 (15.96) |
| Federal State of practice (in medical students: of current training year placement)^j^ |  |  |  |  |  |  |  |
| North Rhine-Westphalia | 42 (32.56) | 22 (32.84) | 20 (32.26) |  | 37 (22.98) | 25 (37.31) | 12 (12.77) |
| Bavaria | 25 (19.38) | 10 (14.93) | 15 (24.19) |  | 20 (12.42) | 4 (5.97) | 16 (17.02) |
| Saxony | 16 (12.40) | 10 (14.93) | 6 (9.68) |  | 3 (1.86) | 0 (0.00) | 3 (3.19) |
| Berlin | 7 (5.43) | 4 (5.97) | 3 (4.84) |  | 4 (2.48) | 2 (2.99) | 2 (2.13) |
| Lower Saxony | 7 (5.43) | 3 (4.48) | 4 (6.45) |  | 19 (11.80) | 4 (5.97) | 15 (15.96) |
| Hamburg | 5 (3.88) | 5 (7.46) | 0 (0.00) |  | 3 (1.86) | 2 (2.99) | 1 (1.06) |
| Hesse | 7 (5.43) | 7 (10.45) | 0 (0.00) |  | 12 (7.45) | 2 (2.99) | 10 (10.64) |
| Saxony-Anhalt | 6 (4.65) | 1 (1.49) | 5 (8.06) |  | 1 (0.62) | 0 (0.00) | 1 (1.06) |
| Schleswig-Holstein | 5 (3.88) | 0 (0.00) | 5 (8.06) |  | 17 (10.56) | 6 (8.96) | 11 (11.70) |
| Baden-Wuerttemberg | 3 (2.33) | 2 (2.99) | 1 (1.61) |  | 13 (8.07) | 9 (13.43) | 4 (4.26) |
| Brandenburg | 3 (2.33) | 1 (1.49) | 2 (3.23) |  | 2 (1.24) | 1 (1.49) | 1 (1.06) |
| Rhineland-Palatinate | 2 (1.55) | 1 (1.49) | 1 (1.61) |  | 6 (3.73) | 1 (1.49) | 5 (5.32) |
| Bremen | 1 (0.78) | 1 (1.49) | 0 (0.00) |  | 2 (1.24) | 1 (1.49) | 1 (1.06) |
| Mecklenburg-Western Pomerania | 0 (0.0) | 0 (0.0) | 0 (0.0) |  | 2 (1.24) | 0 (0.00) | 2 (2.13) |
| Saarland | 0 (0.0) | 0 (0.0) | 0 (0.0) |  | 0 (0.00 %) | 0 (0.00 %) | 0 (0.00 %) |
| Thuringia | 0 (0.0) | 0 (0.0) | 0 (0.0) |  | 2 (1.24) | 0 (0.00) | 2 (2.13) |
| Abroad | - | - | - |  | 18 (11.18) | 10 (14.93) | 8 (8.51) |
| Municipality size of practice (in medical students: Of current training year placement)^k^ |  |  |  |  |  |  |  |
| Rural (up to 10,000 inhabitants) | 23 (17.83) | 10 (14.93) | 13 (20.97) |  | 9 (5.59) | 4 (5.97) | 5 (5.32) |
| Small town (up to 20,000 inhabitants | 19 (14.73) | 8 (11.94) | 11 (17.74) |  | 7 (4.35) | 1 (1.49) | 6 (6.38) |
| Town (more than 20,000 inhabitants) | 37 (28.68) | 21 (31.34) | 16 (25.81) |  | 38 (23.60) | 12 (17.91) | 26 (27.66) |
| City (more than 100,000 inhabitants) | 50 (38.76) | 28 (41.79) | 22 (35.48) |  | 107 (66.46) | 50 (74.63) | 57 (60.64) |
| Setting of practice (in medical students: Of current training year placement)^l^ |  |  |  |  |  |  |  |
| Clinic | 8 (6.20) | 4 (5.97) | 4 (6.45) |  | 155 (96.27) | 65 (97.01) | 90 (95.74) |
| Practice | 111 (86.05) | 59 (88.06) | 52 (83.87) |  | 4 (2.48) | 1 (.49) | 3 (3.19) |
| Medical Care Center (MVZ) | 8 (6.20) | 2 (2.99) | 6 (9.68) |  | 0 (0.0) | 0 (0.0) | 0 (0.0) |
| Other | 2 (1.55) | 2 (2.99) | 0 (0.00) |  | 2 (1.24) | 1 (1.49) | 1 (1.06) |
| Assignment of psychiatric diagnoses^m^ |  |  |  |  |  |  |  |
| Yes | 110 (84.62) | 55 (80.88) | 55 (88.71) |  | 6 (3.70) | 2 (2.94) | 4 (4.26) |
| No | 20 (15.38) | 13 (19.12) | 7 (11.29) |  | 156 (96.30) | 66 (97.06) | 90 (95.74) |
| Standard diagnostic procedure für psychiatric disorders^n^ |  |  |  |  |  |  |  |
| Clinical interview | 10 (7.69) | 5 (7.35) | 5 (8.06) |  | 4 (2.47) | 2 (2.94) | 2 (2.13) |
| Validated checklist | 11 (8.46) | 3 (4.41) | 8 (12.90) |  | 10 (6.17) | 5 (7.35) | 5 (5.32) |
| Screening questionnaire | 45 (34.62) | 26 (38.24) | 19 (30.65) |  | 10 (6.17) | 3 (4.41) | 7 (7.45) |
| Clinical impression | 53 (40.77) | 27 (39.71) | 26 (41.94) |  | 22 (13.58) | 11 (16.18) | 11 (11.70) |
| None | 11 (8.46) | 7 (10.29) | 4 (6.45) |  | 116 (71.60) | 47 (69.12) | 69 (73.40) |

*Note*. Unless otherwise indicated by superscript letters, the response options correspond to the row labels in the table. For further participant details, please refer to **Table 2** in the manuscript. MVZ = Medical Care Center, refers to multidisciplinary outpatient clinics in Germany that provide coordinated medical care by various specialists under one organizational structure.
^a^Item: What is your employment situation? ^b^Item: How many years have you been working as a doctor?/ How many months of training have you received in your current training year? ^c^Item: Which medical field do you currently work in?/ Which medical field are you currently training in? ^d^Item: How many years have you been working in the respective medical field? ^e^Item: How many months have you been trained in your current training year quadrimester placement? ^f^Item: Which semester of your studies are you in? ^g^Item: Are you currently working in primary care?/Are you currently undergoing primary care training? ^h^Item: Do you have work experience in one or more of the fields of psychiatry, psychosomatic medicine, and/or psychotherapy?/ Have you completed a training year quadrimester in one or more of the medical fields of psychiatry, psychosomatic medicine, and/or psychotherapy? ^i^Item: In which medical field would you like to work as a doctor in the future? ^j^Item: In which federal state are you currently working most of the time?/In which federal state are you currently undergoing you current training year placement? ^k^Item: What is the size of the municipality where you currently work?/What is the size of the municipality of your current training year placement? ^l^Item: Which setting are you currently working in?/ Which setting are you currently completing your practical training year placement in? ^m^Item: Do you independently assign F-diagnoses with the specification “confirmed” (without adopting a diagnosis from a clinic/psychotherapist/specialist)? ^n^Item: Do you usually perform a certain diagnostic procedure to assign or exclude (confirmed) F-diagnoses?

**Table S5**

*Intention-to-treat Analyses Coefficient Terms’ Results with Stigma Facet Outcomes as Criteria Per Each Group*

| *Sample* | *DV* | *Coefficient Term* | *Estimate* | *SE* | *t* | *[LL, UL]* |
| --- | --- | --- | --- | --- | --- | --- |
| GPs | Knowledge | Control Group | -0.09 | 0.22 | -0.39 | [-0.51, 0.37] |
|  |  | Timepoint T2 | 0.92 | 0.23 | 4.02 | [0.49, 1.36] |
|  |  | Timepoint T3 | 0.85 | 0.24 | 3.50 | [0.40, 1.30] |
|  |  | Control Group x Timepoint T2 | -0.93 | 0.32 | -2.92 | [-1.59, -0.29] |
|  |  | Control Group x Timepoint T3 | -0.05 | 0.33 | -0.15 | [-0.72, 0.57] |
|  | Affect | Control Group | 0.09 | 0.07 | 1.35 | [-0.04, 0.22] |
|  |  | Timepoint T2 | -0.12 | 0.06 | -1.82 | [-0.24, 0.01] |
|  |  | Timepoint T3 | -0.01 | 0.07 | -0.17 | [-0.15, 0.12] |
|  |  | Control Group x Timepoint T2 | -0.10 | 0.09 | -1.15 | [-0.28, 0.07] |
|  |  | Control Group x Timepoint T3 | -0.26 | 0.09 | -2.77 | [-0.44, -0.07] |
|  | Behavior | Control Group | -0.10 | 0.30 | -0.35 | [-0.69, 0.46] |
|  |  | Timepoint T2 | 1.63 | 0.30 | 5.45 | [1.04, 2.22] |
|  |  | Timepoint T3 | 1.36 | 0.32 | 4.28 | [0.71, 2.01] |
|  |  | Control Group x Timepoint T2 | -2.14 | 0.42 | -5.11 | [-2.89, -1.32] |
|  |  | Control Group x Timepoint T3 | 0.62 | 0.44 | 1.41 | [-0.21, 1.47] |
| Students | Knowledge | Control Group | 0.06 | 0.16 | 0.37 | [-0.27, 0.37] |
|  |  | Timepoint T2 | 0.75 | 0.18 | 4.11 | [0.39, 1.10] |
|  |  | Timepoint T3 | 0.78 | 0.20 | 3.88 | [0.40, 1.20] |
|  |  | Control Group x Timepoint T2 | -1.22 | 0.23 | -5.19 | [-1.70, -0.76] |
|  |  | Control Group x Timepoint T3 | 0.04 | 0.25 | 0.18 | [-0.44, 0.49] |
|  | Affect | Control Group | 0.05 | 0.06 | 0.89 | [-0.06, 0.16] |
|  |  | Timepoint T2 | 0.00 | 0.06 | 0.05 | [-0.11, 0.13] |
|  |  | Timepoint T3 | 0.08 | 0.07 | 1.21 | [-0.04, 0.22] |
|  |  | Control Group x Timepoint T2 | 0.02 | 0.08 | 0.25 | [-0.12, 0.16] |
|  |  | Control Group x Timepoint T3 | -0.23 | 0.08 | -2.70 | [-0.40, -0.06] |
|  | Behavior | Control Group | 0.33 | 0.27 | 1.21 | [-0.19, 0.86] |
|  |  | Timepoint T2 | 2.55 | 0.30 | 8.45 | [1.94, 3.15] |
|  |  | Timepoint T3 | 2.53 | 0.33 | 7.6596146 | [1.89, 3.14] |
|  |  | Control Group x Timepoint T2 | -2.20 | 0.39 | -5.72 | [-2.93, -1.45] |
|  |  | Control Group x Timepoint T3 | -0.43 | 0.41 | -1.04 | [-1.28, 0.42] |

*Note*. Results are from a linear mixed-effects model with random intercepts for participants. Estimates are unstandardized coefficients with standard errors and 95% bootstrapped confidence intervals. LL and UL represent the lower-limit and upper-limit of the confidence interval. DV = Dependent Variable. GPs = General Practitioners. Students = Medical Students. Knowledge = Continuing Medical Education (CME) Knowledge Items. Affect = Opening Minds Stigma Scale for Healthcare Providers (OMS-HC). Behavior = General Self-Efficacy Scale (GSE).
